# Supplementary material for: Using experience-based co-design (EBCD) to develop high-level design principles for a visual identification system for people with dementia in acute hospital ward settings
Source: BMJ Open. 2023 May 10;13(5):e069352. doi: 10.1136/bmjopen-2022-069352 (PMC10174034; doi:10.1136/bmjopen-2022-069352)
Supplement: Supplementary data [file bmjopen-2022-069352supp001.pdf]

PHASE 1 DISCOVERY WORKSHOP	
**Titles of scenario slides**	**Content and questions delivered via actor voice-over**
**Introduction**	Explanation of Visual Identification Systems (VIS)
**Alice's Story**<br>**Alice's VIS**<br>**Alice's hospital**	*What information about Alice is needed to:*<br>• *keep her safe while she is in hospital?*<br>• *help staff communicate well with her?*<br>• *make sure she gets the right treatment and care?*<br>• *avoid agitation and distress?*<br>• *make sure she is treated like a person?*
**Consenting to the identifier**<br>**Issues of Consent**	• *Should patients/relatives give consent for an identifier to be used? If so, what should the care team do to get consent?*<br>• *What do patients/relatives need to know about the implications of having an identifier?*
**George's story**<br>**George's visual IDs**<br>**Continuity of care**	• *How can we make sure that it is clear to all hospital staff what the identifier means, and what they should do when they see it?*<br>• *How can we ensure essential information is easy to find and that all staff know about and consult the information?*
**Claire's story**<br>**Claire's hospital**<br>**Visual identifiers**	• *Given their busy schedules, how can we ensure staff have the time to consult the patient information documents?*<br>• *How can we better ensure that appropriate care continues from one shift to the next?*
**What did we miss?**	• *What did we miss?*

PHASE 2 CO-DESIGN WORKSHOP	
**Scenario slides**	**Content and questions delivered verbally by researchers**
**Recap/intro: VIS**<br>**How it should work**<br><br>**The sign/symbol element**<br>**The information element**<br><br>**How do we overcome these obstacles?**	
**6 hat thinking**	
**The sign / symbol**	• *What should the symbol tell you about the individual?*<br>• *Do different systems across different sites lead to problems?*<br>• *What might make it better?*
	• *What if the symbol was the wristband (perhaps coloured), was personalized and smart in some way - like a unique RIFD, tracking, QR or barcode?*<br>• *What would be the advantages of this approach?*<br>• *What would make this work well?*
**The information (1)**	• *Could a standardised tool be used as part of the admissions process?*<br>• *How best might this information be gathered?*
**The information (2)**	• *What is the key information that is needed to allow them to be treated both as a person and for their specific dementia and other care needs?*<br>• *Would it be best if the essential information about the patient is stored and managed as part of the EPR system, rather than separately stored information?*
**Making the system work (1)**	• *Who would have responsibility for completing this?*<br>• *How could we make sure it is absolutely clear whose responsibility it is to enter the information and that it gets done?*<br>• *How can we make sure that all staff know about, can find, consult and act on this information?*
**Making the system work (2)**	• *Assuming the details are on the system, how would it benefit the bedside care of people with dementia?*<br>• *How can we make this system work when the patient is moved away from the bedside?*
**What did we miss?**	• *What did we miss?*

PHASE 3 VERIFICATION WORKSHOP	
**Stage**	**Content and questions delivered verbally by researchers**
**Welcome and introductions (Plenary)**	
**Recap on progress made in prior workshops (Plenary)**	
**Introduction to the design principles (Plenary)**	• What MUST happen, or MUST be in place, for the system to work so that:<br>1. hospital staff recognise people with dementia more quickly<br>2. respond to their needs more readily<br>3. and provide better, more personalised care
**Sense-checking the design principles (Plenary)**	• Do you have any questions about the principles?<br>• What did you think of the idea of a set of five co-dependent principles?
**Critiquing the design principles (Small mixed groups)**	• Which principle(s) do you most strongly agree with? Why?<br>• Are there any principles on the list that you wouldn't necessarily include? Why?<br>• Are there any missing from the list?
**Feedback (Plenary)**	
**How to implement the design principles (Small mixed groups)**	• How viable is it to implement any/some/all of these principles?<br>• If so, what would need to happen first?<br>• What would help implement them?<br>• What would get in the way?
**Feedback (Plenary)**	
**Anything we missed? (Plenary)**	• *What did we miss?*
