# Supplementary material for: Using experience-based co-design (EBCD) to develop high-level design principles for a visual identification system for people with dementia in acute hospital ward settings
Source: BMJ Open. 2023 May 10;13(5):e069352. doi: 10.1136/bmjopen-2022-069352 (PMC10174034; doi:10.1136/bmjopen-2022-069352)
Supplement: Author's manuscript [file bmjopen-2022-069352.draft_revisions.pdf]

BMJ Open is committed to open peer review. As part of this commitment we make the peer review history of every article we publish publicly available.

When an article is published we post the peer reviewers' comments and the authors' responses online. We also post the versions of the paper that were used during peer review. These are the versions that the peer review comments apply to.

The versions of the paper that follow are the versions that were submitted during the peer review process. They are not the versions of record or the final published versions. They should not be cited or distributed as the published version of this manuscript.

BMJ Open is an open access journal and the full, final, typeset and author-corrected version of record of the manuscript is available on our site with no access controls, subscription charges or pay-per-view fees (http://bmjopen.bmj.com).

If you have any questions on BMJ Open's open peer review process please email info.bmjopen@bmj.com

BMJ Open

Co-designing high-level design principles for a visual identification system for people with dementia in acute hospital ward settings

Journal:	*BMJ Open*
Manuscript ID	bmjopen-2022-069352
Article Type:	Original research
Date Submitted by the Author:	21-Oct-2022
Complete List of Authors:	Macdonald, Alastair; The Glasgow School of Art, School of Design<br>Kuberska, Karolina; University of Cambridge, THIS Institute (The Healthcare Improvement Studies Institute), Department of Public Health and Primary Care<br>Stockley, Naomi; The Point of Care Foundation<br>Fitzsimons, Bev; The Point of Care Foundation
Keywords:	QUALITATIVE RESEARCH, Protocols & guidelines < HEALTH SERVICES ADMINISTRATION & MANAGEMENT, Dementia < NEUROLOGY, Quality in health care < HEALTH SERVICES ADMINISTRATION & MANAGEMENT

SCHOLARONE™
Manuscripts

BMJ

I, the Submitting Author has the right to grant and does grant on behalf of all authors of the Work (as defined in the below author licence), an exclusive licence and/or a non-exclusive licence for contributions from authors who are: i) UK Crown employees; ii) where BMJ has agreed a CC-BY licence shall apply, and/or iii) in accordance with the terms applicable for US Federal Government officers or employees acting as part of their official duties; on a worldwide, perpetual, irrevocable, royalty-free basis to BMJ Publishing Group Ltd ("BMJ") its licensees and where the relevant Journal is co-owned by BMJ to the co-owners of the Journal, to publish the Work in this journal and any other BMJ products and to exploit all rights, as set out in our licence.

The Submitting Author accepts and understands that any supply made under these terms is made by BMJ to the Submitting Author unless you are acting as an employee on behalf of your employer or a postgraduate student of an affiliated institution which is paying any applicable article publishing charge ("APC") for Open Access articles. Where the Submitting Author wishes to make the Work available on an Open Access basis (and intends to pay the relevant APC), the terms of reuse of such Open Access shall be governed by a Creative Commons licence – details of these licences and which Creative Commons licence will apply to this Work are set out in our licence referred to above.

Other than as permitted in any relevant BMJ Author's Self Archiving Policies, I confirm this Work has not been accepted for publication elsewhere, is not being considered for publication elsewhere and does not duplicate material already published. I confirm all authors consent to publication of this Work and authorise the granting of this licence.

For peer review only

TITLE PAGE

Title

Co-designing high-level design principles for a visual identification system for people with dementia in acute hospital ward settings

Alastair S Macdonald *
The Glasgow School of Art
167 Renfrew Street
Glasgow G3 6RQ, Scotland, UK
a.macdonald@gsa.ac.uk
https://orcid.org/0000-0001-9282-6229
tel: 07811-186-717

Karolina Kuberska
THIS Institute (The Healthcare Improvement Studies Institute)
University of Cambridge
Strangeways Research Laboratory
Cambridge CB1 8RN, England, UK
karolina.kuberska@thisinstitute.cam.ac.uk
https://orcid.org/0000-0002-9610-1863

Naomi Stockley
Point of Care Foundation
The Foundry, 17 Oval Way,
London SE11 5RR, England, UK
naomistockley@pointofcarefoundation.org.uk

Bev Fitzsimons
Point of Care Foundation
The Foundry, 17 Oval Way,
London SE11 5RR, England, UK
BevFitzsimons@pointofcarefoundation.org.uk
https://orcid.org/0000-0001-9749-8877

* Corresponding author

KEYWORDS

Patient identification systems, people with dementia, co-design, EBCD, community participation, design principles

WORD COUNT

Excluding abstract, title page, references, figures and tables: 2870

For peer review only

ABSTRACT

Objectives

Using codesign to improve the quality of care is a well-established approach in healthcare improvement. We tested a modified co-design process to develop a set of high-level design principles for visual identification systems (VIS) for hospitalised people with dementia. Routinely used in UK hospitals for people with dementia, these systems help staff quickly recognise additional needs and adapt their care accordingly. None of the multiple VIS in use in the UK has been rigorously evaluated or been subject to systematic development informed by design principles. We designed and ran remote workshops in three phases with carers of people with dementia and healthcare staff. In phase 1 we presented participants with scenarios based on findings from prior research, prompting participants to discuss their own experiences of VIS. Phase 2 used more future-focused scenarios, prompting participants to co-design improved VIS. In phase 3, a set of provisional design principles developed from our analysis of phases 1 and 2 data were discussed.

Setting

Due to pandemic restrictions, our workshops with carers and healthcare staff were held online.

Participants

A total of 26 carers, and 9 staff took part in a pilot and three separate workshops.

Results

We identified a set of six dementia-friendly design principles for improving the effectiveness of VIS. Participants suggested that, in addition to the use of an identifier and key personal information, professional standards training, effective information and records management, and improved means to involve carers and/or families were key to the effective operation of VIS, leading us to expand a narrow understanding of a VIS. Using scenario-led co-design approach can help trigger useful discussions with staff and carer groups, identify current problems with VIS, and develop a set of high-level design principles for their improvement. These principles reveal day-to-day frictions that require further attention and resolution.

STRENGTHS AND LIMITATIONS OF OUR STUDY

- Designing and running a programme of online workshops using scenario-based narratives is feasible and may be a means of reducing some of the time, cost, and accessibility implications of holding in-person workshops.
- Presenting narratives on slide shows with voice-over can effectively replace videoed witness accounts typically used in EBCD studies.
- EBCD approach can be used in evaluation studies focused on abstract, high level design, such as principles for visual identification systems.
- The study would have benefited from a broader representation of healthcare professionals including, e.g., ward managers, health care assistants, out-patients' department staff, and peripatetic staff such as porters.
- Future studies should involve, with appropriate support, patients with dementia and their carers.

For peer review only

INTRODUCTION

People affected by dementia and some other forms of cognitive impairment in hospital settings face a range of risks that present important challenges for care. One possible intervention to support care is what is commonly referred to as a visual identification system (VIS). This can help staff recognise people with cognitive impairment quickly and easily and adapt their care accordingly. VIS have normally been understood as having two key elements, an ‘identifier’ and ‘information’. The identifier is usually an easily noticeable sign or symbol, such as a specially coloured wristband, flower or butterfly.(1,2) The presence of the identifier signifies to staff that further important information about the patient’s care and personal needs should be consulted and appropriate action taken; this additional information can be made readily available via patient profile documents, e.g., a patient passport,(3) ‘This is me’ booklet,(4) ‘About Me’ records,(5) or bedside poster. Although VIS are broadly used within the NHS, some issues have been identified.(6) Currently, a number of localised versions of VIS are in use, but none has been rigorously evaluated or subject to systematic development informed by dementia-friendly design principles.

The DA VINCI (Developing A Visual IdeNtification method for people with Cognitive Impairment in institutional settings) programme looked at the use of VIS for people with dementia in hospital settings, with a view to identifying an existing system that might be further developed or, alternatively, co-designing a new system around the needs and preferences of patients, carers and health professionals to ensure acceptability and workability in practice.(7) DA VINCI took a multi-disciplinary approach involving four linked studies from different organisations and institutions. These comprised: a survey of current practice around the visual identification of people with cognitive impairment in hospital settings;(8) a desk-based analysis of the ethical and legal issues involved in the use of such systems;(9) a qualitative study, including in-depth case studies of and interviews about current applications of VIS in hospitals with staff, people with dementia and their carers; and a co-design study comprising a series of participatory workshops with carers and staff to co-develop a set of design principles. This paper describes the co-design study, the high-level principles identified and how these were produced.

Co-design

Experience-based co-design (EBCD) has been used to achieve quality improvements in a range of healthcare settings by bringing staff and patients together through a six-stage (or accelerated five-stage) process structured within a single study.(10,11) Examples include improvements to a mental health inpatient service, and improving the pre-treatment care pathway for cancer patients.(12,13) Normally, in the early ‘discovery’ phase of EBCD, trigger films, i.e. videoed witness accounts of particular issues relating to the service in question, are created to prompt discussion at separate, and then joint, staff and patient events.(14) Staff and patient groups then come together during a later ‘co-design’ stage to suggest improvements to healthcare delivery and experience. We modified the EBCD approach given the separate study set-up in the DA VINCI programme, relying on the findings of the qualitative study for our discovery phase. Also, due to restrictions imposed by the pandemic, the option of creating trigger films to prompt discussion was not open to us. Instead, we created a set of scenario-based narratives relying on the findings of the qualitative study and prior experiences of our study team. COVID-19 rules also necessitated a move to online rather than face-to-face workshops.

For peer review only

METHODS

Materials: scenario-based narratives

We prepared a series of text documents describing short scenarios facing a fictional patient with dementia (Alice) admitted to hospital. These highlighted several dementia-related issues particular to Alice at a hospital-, ward-, and patient-level, interspersed with key questions around issues that potentially affected Alice's care where a VIS might have an important role to play. Two further sets of scenarios were similarly developed, for patient George with Lewy bodies dementia and ward manager Claire. All three were translated into an illustrated slideshow format with added actor voice-overs (Supplemental files 1a-f, 2). Given the variety of VIS in use in UK hospitals, the scenarios and questions were intentionally generic rather than specific to any single VIS and were designed to support a structured online workshop of about two hours' duration.

Study design

These scenarios were piloted in a workshop for carers. There, participants found it difficult to shift from pre-occupations with their experiences of existing VIS to think more speculatively about improvements. We therefore decided to hold the workshops in two separate phases within a week of one another. Phase 1 workshops (discovery) focused on identifying critical issues with the status quo; phase 2 allowed for more speculative thinking to improve these.(15) The phase 1 scenarios covered: i) the frail older patient Alice, her VIS, being in hospital, and issues of consent for the use of the VIS; ii) patient George, and his care while being moved out of the ward and around the hospital; and iii) ward manager Claire, responsible for ensuring staff consulted relevant information and provided good continuity of person-centred care. The phase 2 scenarios focussed on improving three specific aspects: i) the identifier; ii) the information; and iii) how the VIS could function better. Phase 1 and phase 2 workshops were held with carers and staff separately; all participants were sent a copy of the slides prior to these workshops for their consideration and reference. Following analysis of data from these two phases, we drafted a set of provisional design principles, which we brought back to a meeting of the DA VINCI study partners. The phase 3 workshop brought both carers and staff together to discuss these. This reviewed set of principles and feedback were then brought to a meeting of our Expert Collaborative Group (ECG) for discussion and consolidated after addressing their feedback. Table 1 summarises the study design.

For peer review only

Date	Phase	Workshop participants	Participant numbers	Materials used	Questions explored	Detail
Dec-20	Design development workshop with Expert Collaborative Group					
Mar-21	Proposal for use of scenarios and study design discussed with Expert Collaborative Group					
Oct-21	**Pilot**	Carers	5	Status quo scenarios	Issues with the status quo	Supplemental materials 1a-f, 2
Nov-21	**Phase 1**	Staff	2	Status quo scenarios	Issues with the status quo	Supplemental materials 1a-f, 2
		Carers	7			
Nov-21	**Phase 2**	Staff	3	What if scenarios	Suggestions for improvement	Supplemental material 3
		Carers	7			
Nov-21>Apr-22	Analysis of phase 1 and 2 data					Worked example Table 2
Feb-22>Apr-22	Formulation of proposed design principles					Supplemental materials 4, 5
May-22	Proposed design principles discussed with DA VINCI programme partners					Supplemental materials 4, 5
May-22	**Phase 3**	Joint staff and carers	11 *4 staff, 7 carers*	Design Principles Version 1	Viability of Design Principles	Supplemental materials 4, 5
Jul-22	Proposed design principles discussed with Expert Collaborative Group					Supplemental materials 4, 5

Table 1. Study design.

For peer review only

Staff and carer participants

Healthcare staff contributed to the study in a variety of ways: through participation in the qualitative study; through our ECG; and by taking part in our workshops. They represented a varied range of expertise including old age psychiatry and specialist dementia nursing. Carer participants were identified through the charity Dementia Carers Count. There was a range of expertise among the carers: length of time caring; type and severity of the dementia being cared for; setting in which care is/was provided; and experiences of hospital care, and the variability of hospital routines. All participants were reimbursed for their time in accordance with INVOLVE guideline.(16)

Data collection and initial analysis

The qualitative analysis was guided by the pragmatic theoretical framework, focusing on obtaining a practical understanding of real-world issues.(17) Researchers AM, BF and NS gathered field notes during all workshops including, wherever possible, verbatim remarks. Each researcher independently coded their field notes and identified the key themes. These were then compared, and a consensus on the key themes was reached between the researchers. All workshops were video recorded, and if there were areas of lack of clarity or agreement about the key themes, the recordings were consulted to help resolve the issue. We analysed our field notes focusing on recurrent themes and patterns of experiences identified from field notes or paraphrasing common ideas from the conversations that took place in workshops. We describe the process of drafting and refining the co-design principles below.

Patient and Public Involvement

No patients were involved in the design, conduct, reporting, or dissemination plans of this study. Due to the pandemic, workshop participants did not include people with dementia, but any future studies would involve, with appropriate support, people with dementia and their carers as their experience should be key to nuancing the design principles of a VIS. The study teams were assisted by our ECG composed of healthcare professionals working with patients with dementia, carers for people with dementia, and third sector representatives, who were invited to comment on the methodology and findings of the studies throughout the project. Our initial study plans were shared with the ECG at the outset, the detailed methodology was shared mid-way through the project, ahead of the delivery of the workshops, and the ECG were invited to comment on our first draft design principles. The results of the study have been disseminated to all staff and carer participants, partner study teams and the ECG.

RESULTS

We coalesced recurrent subthemes into distinct, identifiable themes, highlighting the key issues. Examples of the key themes identified from the coalescence of points arising from our workshop notes are shown and summarised in Table 2.

Theme	Subtheme	Key points identified from workshop notes
Training in dementia care	Lack of dementia expertise<br><br>Lack of awareness of relevance of the symbol	Experiential training helps staff really understand what it's like to have dementia<br><br>The UK Core Skills Training Framework includes dementia awareness training for every member of staff. Tier 2 training is required for every patient-facing member of staff.<br><br>Training sessions need to be more frequent; many staff members don't have up-to-date training.
The identifier and links to information	Lack of consistency between hospitals	Information about diagnosis needs to link to alert on the Electronic Patient Records (EPR).

For peer review only

	No link to data	Linking primary care records with hospital records such that if a person with dementia presents to hospital, specialist dementia service is notified.<br><br>Critical information should be presented succinctly (not 4-5 pages).
Information	Information should be relevant, succinct, and easy to access	Include key pieces of information about the person (preferences for name, physical contact, food, LPA, DNA, etc.) and ways to contact family and carers.<br><br>Patient profile documents would benefit from a redesign towards presenting key information in a succinct way.
Family participation	Input of family is vital<br><br>Involving family to establish patient preferences and legal provisions.	Routinely contacting family and carers, especially when contact with patient is difficult to avoid making assumptions about legal provisions (e.g. Do-Not-Resuscitate orders).
Standardisation	Ensuring standard of care	Expectation that patients receive similar levels of care irrespective of which hospital they stay at.

Table 2. Themes, subthemes and key points identified from workshop notes.

Training

Participants often mentioned the need for regular, ongoing and professional standards training. Of particular concern was high staff turnover and the challenges with ensuring consistency of understanding and communication, including using positive language when discussing patients with dementia, across both regular and bank staff. The need for training also applied to other non-clinical staff, such as porters. Carers emphasised the importance of an institutional leadership and organisational culture. They also highlighted that experiential training sessions can be more effective for staff, as healthcare professionals are more likely to retain the information if they are learning by doing. There was consensus that the training needed to be of sufficient quality and should not be a tick-box exercise – recognising that mandatory training, especially online, can be of poor quality.

Linking the identifier to the key personal information

Participants pointed out the problems arising from an individual's key personal information not accompanying them around various hospital departments or community services. Some saw the solution in technology, such as smart-linking key personal information to the identifier (e.g., through a QR or bar code), so that this would be readily available at each and every point in the patient care pathway. Some participants saw value in relevant summary information about the patient being available in a physical form as well, suggesting that there may be situations where staff might not have time or opportunity for an extra step of scanning a code or checking the electronic patient record.

Integration and management of records

Participants often mentioned that key, vital medical and personal information often exists in separate (e.g., in GP, hospital, community) electronic and paper-based systems, which are not integrated with each other. Participants felt that VIS could be instrumental to much better information integration and management along the whole patient care pathway, to include key search functions, automated alerts and links to that individual's care support needs. Again, many suggested that technology may offer useful solutions, from wearable technology to individual devices with screens at patient bedside.

For peer review only - http://bmjopen.bmj.com/site/about/guidelines.xhtml

Enabling carers and families to provide key information

Participants highlighted the need for up-to-date, readily available, accessible and prioritised key information about each hospitalised individual with dementia, e.g., name preference, food preferences, allergies, ability to communicate and/or give consent, Lasting Power of Attorney, and Do-Not-Resuscitate orders, to enable appropriate, personalised care. Relatives and carers felt, where possible, they should be enabled to input and update key personal information and emphasised the importance of being able to connect to their family member via a video call, similar to the Relatives Gateway online portal in use in some care homes.

Standardising the identifier

Perhaps the most contentious issue to arise relating to the identifier was what participants referred to as "standardisation". Many workshop participants considered that a standardised, nationally mandated identifier, applied on admission with assumed consent (unless revoked), might provide greater consistency in recognising patients across different trusts and hospitals with a confirmed diagnosis of dementia, particularly given staff mobility across different sites using different systems. However, our participants who were healthcare professionals had concerns about how difficult it would be to implement a unified system across the whole NHS given that different VIS are already in existence.

DESIGN PRINCIPLES

Plsek et al. state that '[d]esign rules are heuristic statements in the form: If you want to achieve outcome Y in situation S, something like X might help.'(18) Our approach to deriving design principles (using the term principles in preference to rules) from the Phase 1 and 2 thematic analysis was adapted from their concept which 'helps convert the tacit knowledge of organizational change agents into explicit, actionable knowledge'.(18) We used an adapted version of their methods 3 and 4 involving storytelling to translate our findings, through a number of iterations, into a set of draft principles for the joint phase 3 workshop. We provided these in concise form (see SF5) to all participants in a single sheet which we supplemented with additional explanations at the workshop with further expansion on the principles (see SF6). We invited participants to comment on these individually in small breakout groups and at plenary sessions. We asked: 'If these principles were adopted, would: hospital staff recognise people with dementia more quickly; respond to their needs more readily; and provide better, more personalised care?' BF, NS and AM took notes of the discussion.

In the phase 3 workshop and ECG feedback, participants generally agreed that some of the principles would be more problematic or take longer to implement than others, e.g., improved IT systems where the various clinical records, such as from hospital, GP, care home and memory clinic records, could be better integrated with readily accessible key personal information. The point was also made that relying purely on electronic records was risky and that supplementary paper-based information would be essential. When asked which of the principles would be more fundamentally important to implement, phase 3 participants debated two in particular: training (contingent on organisational culture and leadership) and the form of the identifier (a wristband, symbol etc.).

Importantly, opinions were divided over which principle was more important. Some participants thought that a national, standardised identifier would be relatively easy to implement, especially with third-sector support for disseminating the identifier. However, as pointed out by the ECG, given how well-established the currently used systems already are, a single nationally mandated identifier would be difficult, if not impossible, to implement. With regards to training, inadequately trained staff and lack of an ongoing training programme for all patient-facing employees, particularly for new or agency staff, could lead to them not

For peer review only

being able to interpret the identifier in a way that supports patients as individuals. On a number of occasions, training was referred to as the 'bedrock principle'.

A key point from both the phase 3 workshop and the subsequent ECG discussion highlighted the interdependency of the principles which, if adopted singularly and piecemeal, would be unlikely to achieve the goal of improved, more consistent, personalised care, in hospital, for people with dementia. In other words, these principles need to be implemented systemically and as a set, if their benefits are to be realised. As a result, we added the interdependency of principles as a further principle. The feedback we received from phase 3, the ECG, and the project partners helped refine further our principles into a second version. The challenge involves making the principles simple yet adaptable so that they can serve a large, varied population in the most effective way possible.

DISCUSSION

Confronting high-level principles with reality

In phases 1 and 2, we focussed on the more tangible elements of the VIS – the identifier and the key information and how these might be made to work better, which in turn provoked responses about issues concerning the less visible elements, leading to our findings. We acknowledge that formulating our findings into principles using Plsek et al.'s heuristic was challenging for us (requiring several iterations) and that these were not fully resolved in their initial form in the phase 3 workshop. None-the-less, these principles served as a means of engaging participants in a more abstract level of discussion, along the lines of 'If we were to propose a set of design principles such as these, what would be your response be?'. Participants readily engaged with this more abstract form of discussion.

The value of the phase 3 and ECG feedback to our draft principles was to reveal the 'sticky materiality of practical encounters',(19) i.e. the potential gap between high-level principles and the friction-laden character of situations on the ground, as well as the need for designing around what happens in reality around how identifiers for people with dementia are used in practice. It also highlighted the importance of a participatory process, whereby a proposed improvement, in prototype form (in this case our draft principles), can be interrogated and iteratively refined.

Broadening the understanding of an effective VIS

While the two elements – the identifier and some form of document holding key personal information – are undeniably important, it became clear that for a VIS to function effectively, other elements – as identified in our findings – play a vital role in a functioning of the system that benefits the patient with dementia.

CONCLUSION

Our study shows that an adapted co-design approach using scenarios can trigger useful discussions with staff and carer groups, identify problems with VIS, and engage participants in an abstract discussion about high-level design principles and how these might guide the improvement of VIS.(20) While we recognise these principles are not yet fully resolved, we have made progress towards better defining these principles. Constraints on the normal EBCD approach imposed by the linked-study commissioning method and by the pandemic as detailed above proved an initial challenge; ultimately, they were not an impediment to creatively developing an effective approach sufficient to yield findings challenging our current conception of VIS.

For peer review only

Acknowledgements

We acknowledge the helpful feedback, at all stages in our study from Graham Martin, Director of Research at THIS Institute, and at key points, from our Expert Collaborative Group. Actor Francesca Isherwood provided the voice-overs for the Phase 1 materials. Joanna Goodrich presented the study with NS to the HSRUK conference in Sheffield in July 2022. We gratefully acknowledge the participation of the carers and staff in the workshops, and members of the Expert Collaborative Group for their constructive input. We are grateful to the charity Dementia Carers Count for connecting us with the carers included in this study.

Contributor Statement

BF, NS and AM were the researchers, led by BF, comprising the co-design study and all contributed to the drafting of this manuscript, led by AM. KK, reviewed and substantially edited and contributed to the manuscript. All authors reviewed the final version.

Competing Interests

The authors declare no conflict of interest. The funders had no role in the design of the study; in the collection, analyses or interpretation of data; in the writing of the manuscript, or in the decision to publish the results.

Funding

Health Foundation, Grant/Award Number: RHZF/001 - RG88620; Health Services and Delivery Research Programme, Grant/ Award Number: NF-SI-0617-10026

Data Sharing Statement

Not applicable. Hand-written notes only taken for later thematic analysis.

Ethical Approval Statement

As service improvement, this EBCD study did not require an ethics review.

REFERENCES

1. Wye Valley NHS Trust. Forget me not [Internet]. Herefordshire Council; 2018 [cited 2022 Sep 8]. Available from: https://www.wyevalley.nhs.uk/visitors-and-patients/county-hospital-(acute)/in-patients/dementia-care/forget-me-not-1.aspx

2. The Butterfly Scheme. The Butterfly Scheme | Reaching out to people with dementia and to anyone needing memory support [Internet]. 2017 [cited 2022 Sep 8]. Available from: https://butterflyscheme.org.uk/

3. East Sussex Healthcare NHS Trust. This is me care passport [Internet]. 2013 [cited 2022 Sep 8]. Available from: https://www.esht.nhs.uk/wp-content/uploads/2017/10/This-is-me-My-Care-Passport.pdf

4. Alzheimer's Society. This is me [Internet]. 2022 [cited 2022 Sep 8]. Available from: https://www.alzheimers.org.uk/get-support/publications-factsheets/this-is-me

5. Professional Records Standards Body. About me [Internet]. 2022 [cited 2022 Sep 8]. Available from: https://theprsb.org/standards/aboutme/

6. Featherstone K, Boddington P, Northcott A. Using Signs and Symbols to Label Hospital Patients with a Dementia Diagnosis: Help or Hindrance to Care? Narrative Inquiry in Bioethics. 2020;10(1):49–61.

For peer review only - http://bmjopen.bmj.com/site/about/guidelines.xhtml

7. THIS Institute. Developing a visual identification method for people with cognitive impairment in institutional settings [Internet]. THIS Institute - The Healthcare Improvement Studies Institute. 2020 [cited 2022 Sep 8]. Available from: https://www.thisinstitute.cam.ac.uk/research-projects/davinci/

8. Kuberska K, Dixon-Woods M, Martin G. Visual identifier systems for patients with cognitive impairment in healthcare settings: a survey of practice in UK hospitals. International Journal of Older People Nursing. 2022;

9. Brigden T, Mitchell C, Ordish J, Hall A. Visual identifiers in the care of people with dementia: An ethical and legal analysis [Internet]. Cambridge: PHG Foundation; 2020 [cited 2022 May 9]. Available from: https://www.phgfoundation.org/media/44/download/visual-identifiers-in-the-care-of-people-with-dementia.pdf?v=1&inline=1

10. Point of Care Foundation. EBCD: Experience-based co-design toolkit [Internet]. Point of Care Foundation. n.d. [cited 2022 Sep 8]. Available from: https://www.pointofcarefoundation.org.uk/resource/experience-based-co-design-ebcd-toolkit/

11. Goodrich J. Why experience-based co-design improves the patient experience. The Journal of Health Design. 2018 Mar 22;3(1):84–5.

12. Springham N, Robert G. Experience based co-design reduces formal complaints on an acute mental health ward. BMJ Open Quality. 2015 Jan 1;4(1):u209153.w3970.

13. Brady GC, Goodrich J, Roe JWG. Using experience-based co-design to improve the pre-treatment care pathway for people diagnosed with head and neck cancer. Support Care Cancer. 2020 Feb;28(2):739–45.

14. Locock L, Robert G, Boaz A, Vougioukalou S, Shuldham C, Fielden J, et al. Using a national archive of patient experience narratives to promote local patient-centered quality improvement: an ethnographic process evaluation of “accelerated” experience-based co-design. J Health Serv Res Policy. 2014 Oct;19(4):200–7.

15. Dunne A, Raby F. Speculative everything: design, fiction, and social dreaming. Cambridge, Massachusetts ; London: The MIT Press; 2013. 224 p.

16. INVOLVE, NIHR. Policy on payment of fees and expenses for members of the public actively involved with INVOLVE [Internet]. 2016 [cited 2022 Sep 8]. Available from: https://www.invo.org.uk/wp-content/uploads/2016/05/INVOLVE-internal-payment-policy-2016-final-1.pdf

17. Ramanadhan S, Revette AC, Lee RM, Aveling EL. Pragmatic approaches to analyzing qualitative data for implementation science: an introduction. Implement Sci Commun. 2021 Jun 29;2(1):70.

18. Plsek P, Bibby J, Whitby E. Practical Methods for Extracting Explicit Design Rules Grounded in the Experience of Organizational Managers. The Journal of Applied Behavioral Science. 2007 Mar 1;43(1):153–70.

19. Tsing AL. Friction: An Ethnography of Global Connection. Princeton: Princeton University Press; 2005. 336 p.

For peer review only

20. Fitzsimons B. The challenges of using feedback from patients to inform design. The Journal of Health Design; Vol 3, No 2 (2018): Using patient feedback to inform healthcare design [Internet]. 2018; Available from: https://www.journalofhealthdesign.com/JHD/article/view?path=

For peer review only

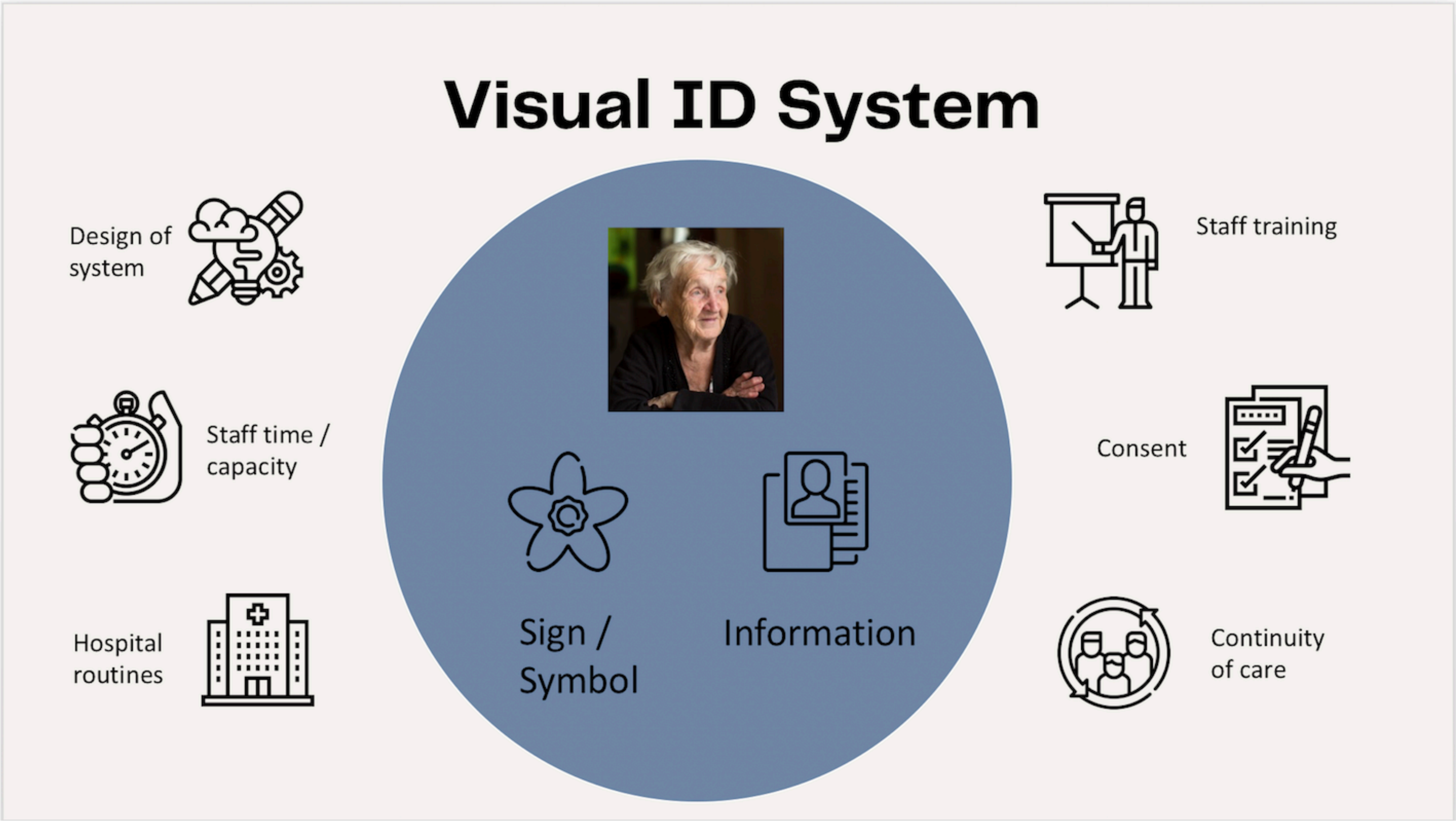
Visual ID System
Design of system
Staff time / capacity
Hospital routines
Sign / Symbol
Information
Staff training
Consent
Continuity of care

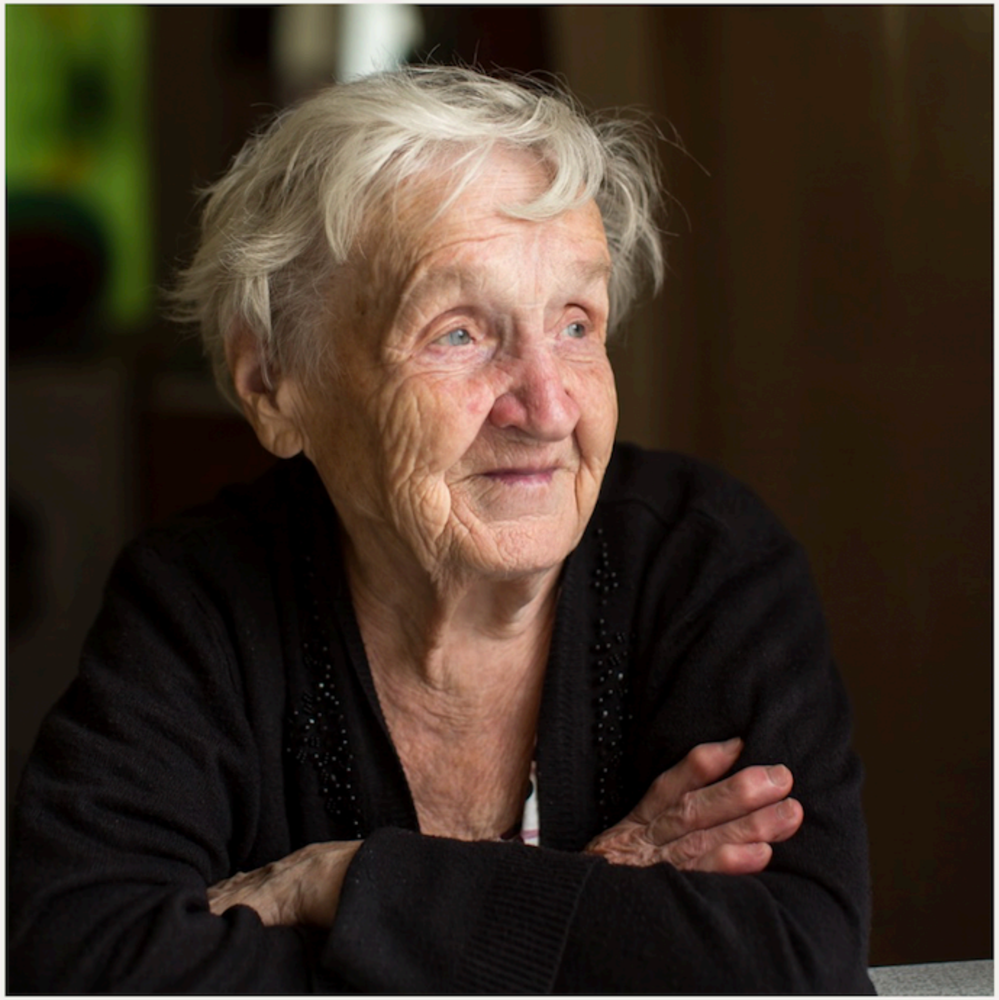

Listen here

Alice's Story

Feel free to use your workbooks to make notes as you listen.

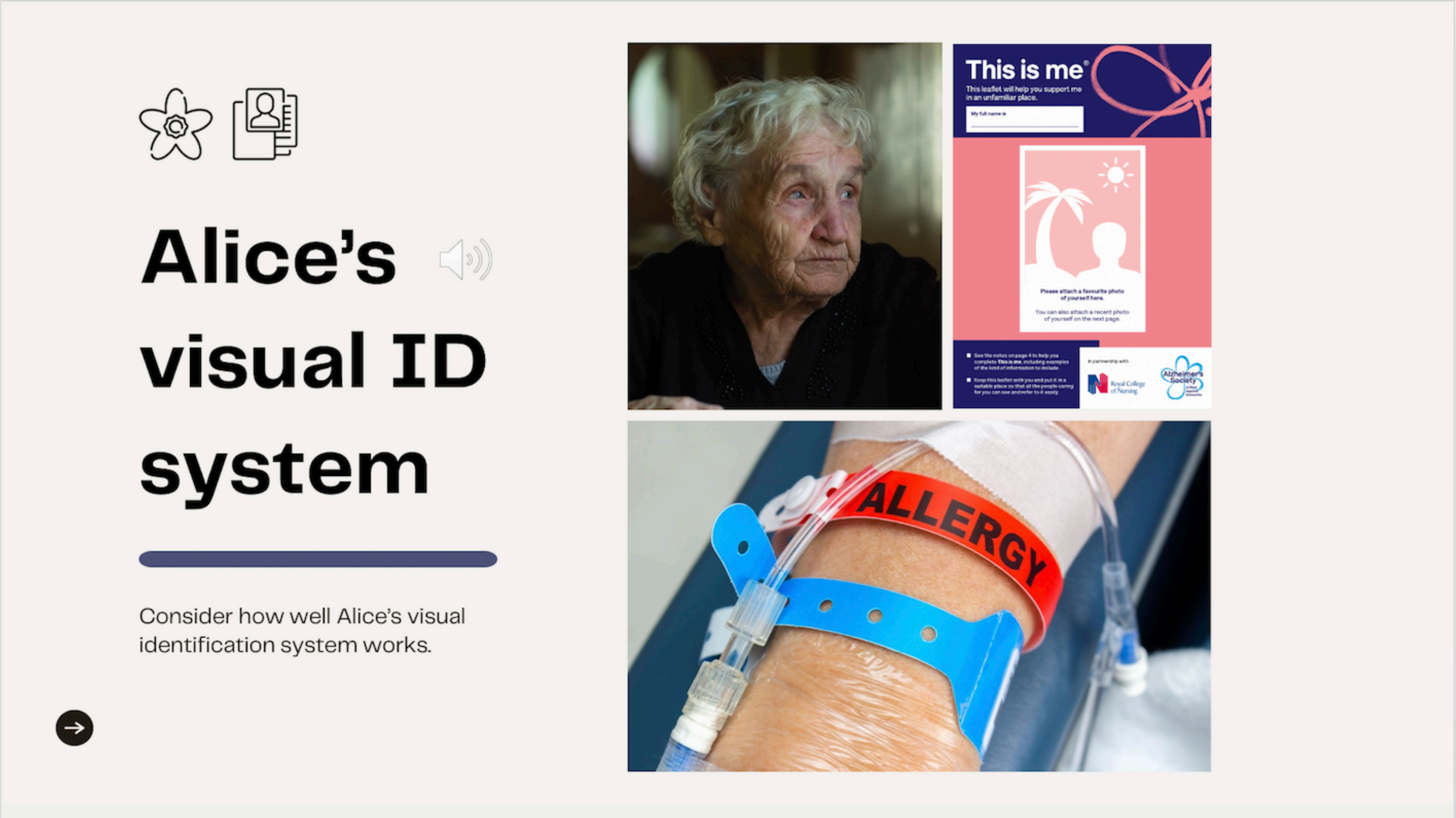
Alice's visual ID system
Consider how well Alice's visual identification system works.
This is me
This leaflet will help you support me in an unfamiliar place.
My full name is
Please attach a favourite photo of yourself here.
You can also attach a recent photo of yourself on the next page.
In partnership with
Royal College of Nursing
Alzheimer's Society
ALLERGY

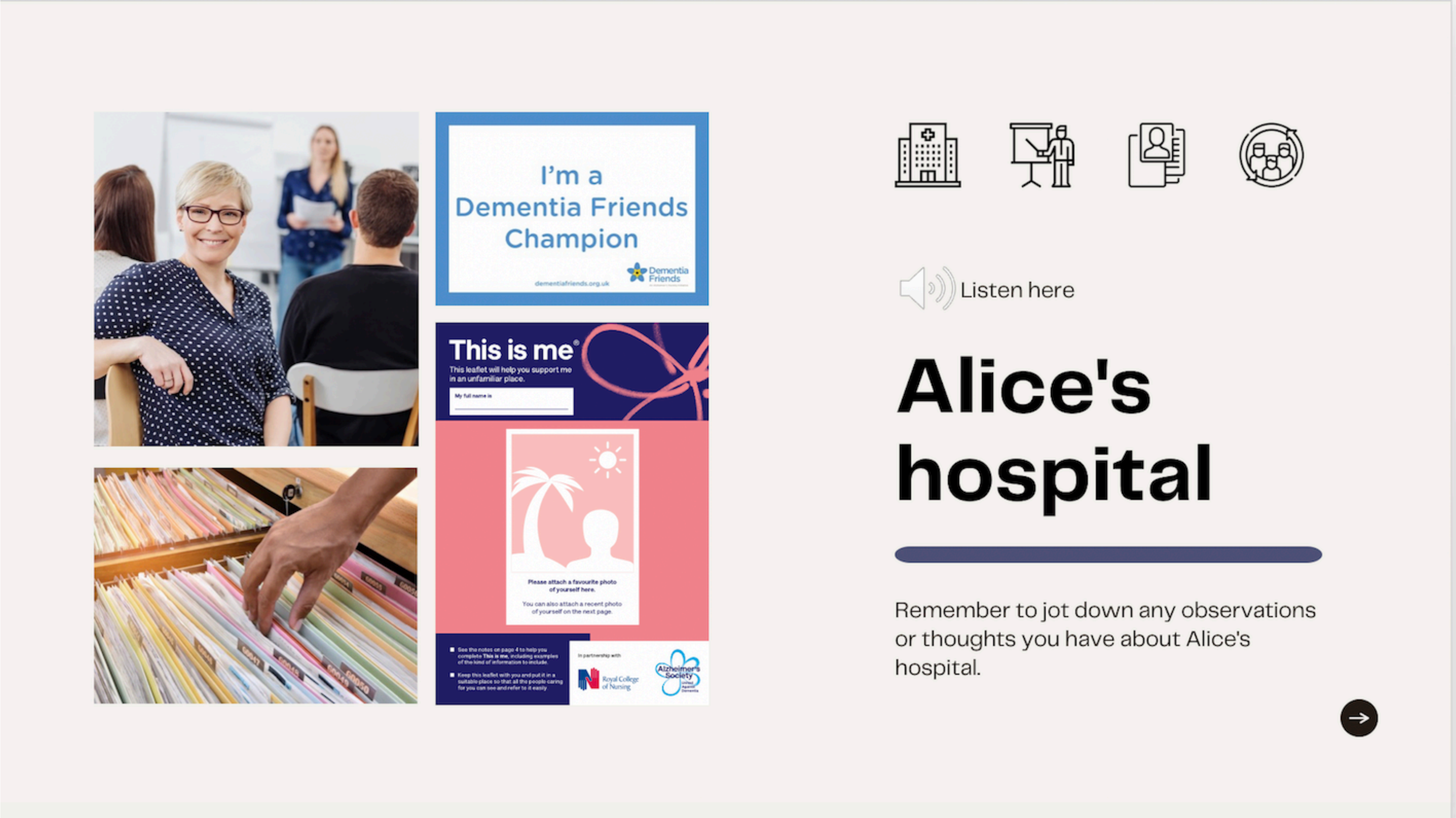
I'm a Dementia Friends Champion
Dementia Friends
This is me
This leaflet will help you support me in an unfamiliar place.
Please attach a favourite photo of yourself here.
Royal College of Nursing
Alzheimer's Society
Listen here
Alice's hospital
Remember to jot down any observations or thoughts you have about Alice's hospital.

Over to you

01 What information about Alice is needed to:

a) keep her safe while she is in hospital?

b) help staff communicate well with her?

c) make sure she gets the right treatment and care?

d) avoid agitation and distress?

e) make sure she is treated like a person?

For peer review only - http://bmjopen.bmj.com/site/about/guidelines.xhtml

Over to you

03/11/21

02 Should patients/relatives give consent for an identifier to be used? If so, what should the care team do to get consent?

03 What do patients/relatives need to know about the implications of having an identifier?

Suppl. 2: Summary of Phase 1 discovery workshop structure and questions exploring recent experiences of VIS

PHASE 1 DISCOVERY WORKSHOP	
**Titles of scenario slides**	**Content and questions delivered via actor voice-over**
**Introduction**	Explanation of Visual Identification Systems (VIS)
**Alice's Story**<br>**Alice's VIS**<br>**Alice's hospital**	*What information about Alice is needed to:*<br>• *keep her safe while she is in hospital?*<br>• *help staff communicate well with her?*<br>• *make sure she gets the right treatment and care?*<br>• *avoid agitation and distress?*<br>• *make sure she is treated like a person?*
**Consenting to the identifier**<br>**Issues of Consent**	• *Should patients/relatives give consent for an identifier to be used? If so, what should the care team do to get consent?*<br>• *What do patients/relatives need to know about the implications of having an identifier?*
**George's story**<br>**George's visual IDs**<br>**Continuity of care**	• *How can we make sure that it is clear to all hospital staff what the identifier means, and what they should do when they see it?*<br>• *How can we ensure essential information is easy to find and that all staff know about and consult the information?*
**Claire's story**<br>**Claire's hospital**<br>**Visual identifiers**	• *Given their busy schedules, how can we ensure staff have the time to consult the patient information documents?*<br>• *How can we better ensure that appropriate care continues from one shift to the next?*
**What did we miss?**	• *What did we miss?*

For peer review only

Suppl 3: Summary of Phase 2 co-design workshop structure and questions exploring potential improvements to VIS

PHASE 2 CO-DESIGN WORKSHOP	
**Scenario slides**	**Content and questions delivered verbally by researchers**
**Recap/intro: VIS**<br>**How it should work**<br><br>**The sign/symbol element**<br>**The information element**<br><br>**How do we overcome these obstacles?**	
**6 hat thinking**	
**The sign / symbol**	• *What should the symbol tell you about the individual?*<br>• *Do different systems across different sites lead to problems?*<br>• *What might make it better?*
	• *What if the symbol was the wristband (perhaps coloured), was personalized and smart in some way - like a unique RIFD, tracking, QR or barcode?*<br>• *What would be the advantages of this approach?*<br>• *What would make this work well?*
**The information (1)**	• *Could a standardised tool be used as part of the admissions process?*<br>• *How best might this information be gathered?*
**The information (2)**	• *What is the key information that is needed to allow them to be treated both as a person and for their specific dementia and other care needs?*<br>• *Would it be best if the essential information about the patient is stored and managed as part of the EPR system, rather than separately stored information?*
**Making the system work (1)**	• *Who would have responsibility for completing this?*<br>• *How could we make sure it is absolutely clear whose responsibility it is to enter the information and that it gets done?*<br>• *How can we make sure that all staff know about, can find, consult and act on this information?*
**Making the system work (2)**	• *Assuming the details are on the system, how would it benefit the bedside care of people with dementia?*<br>• *How can we make this system work when the patient is moved away from the bedside?*
**What did we miss?**	• *What did we miss?*

For peer review only

Suppl 4. Summary of Phase 3 workshop structure and questions used to probe initial findings

**PHASE 3 VERIFICATION WORKSHOP**	
**Stage**	**Content and questions delivered verbally by researchers**
Welcome and introductions (Plenary)	
Recap on progress made in prior workshops (Plenary)	
Introduction to the design principles (Plenary)	• What MUST happen, or MUST be in place, for the system to work so that:<br>1. hospital staff recognise people with dementia more quickly<br>2. respond to their needs more readily<br>3. and provide better, more personalised care
Sense-checking the design principles (Plenary)	• Do you have any questions about the principles?<br>• What did you think of the idea of a set of five co-dependent principles?
Critiquing the design principles<br>(Small mixed groups)	• Which principle(s) do you most strongly agree with? Why?<br>• Are there any principles on the list that you wouldn't necessarily include? Why?<br>• Are there any missing from the list?
Feedback (Plenary)	
How to implement the design principles (Small mixed groups)	• How viable is it to implement any/some/all of these principles?<br>• If so, what would need to happen first?<br>• What would help implement them?<br>• What would get in the way?
Feedback (Plenary)	
Anything we missed? (Plenary)	

For peer review only

Towards a smarter Visual Identification System?

**PRINCIPLE**		**EXAMPLE OF MEANS TO ACHIEVE THIS**
1. The hospital/trust provides a professionally-trained workforce and an appropriate culture of care	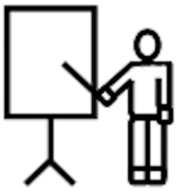	Institutional culture and training
2. The symbol is standardised	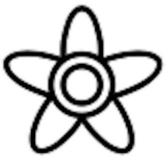	National mandate
3. Key personal Information is readily available and accessible	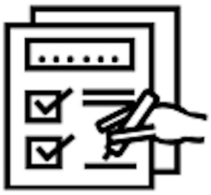	
4. Relatives and carer are involved in providing key information and monitoring care	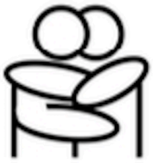	Relatives gateway
5. Key personal information is integrated into the Electronic Patient Record (EPR)	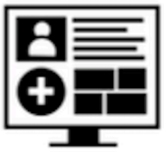	Smart, automated EPR system

If these principles were adopted, would they help staff recognize people with dementia more quickly, respond to their needs more readily, and provide better, more personalised care?

BMJ Open

Using experience-based co-design (EBCD) to develop high-level design principles for a visual identification system for people with dementia in acute hospital ward settings

Journal:	*BMJ Open*
Manuscript ID	bmjopen-2022-069352.R1
Article Type:	Original research
Date Submitted by the Author:	21-Feb-2023
Complete List of Authors:	Macdonald, Alastair; The Glasgow School of Art, School of Design<br>Kuberska, Karolina; University of Cambridge, THIS Institute (The Healthcare Improvement Studies Institute), Department of Public Health and Primary Care<br>Stockley, Naomi; The Point of Care Foundation<br>Fitzsimons, Bev; The Point of Care Foundation
**Primary Subject Heading**:	Qualitative research
Secondary Subject Heading:	Health services research, Patient-centred medicine
Keywords:	QUALITATIVE RESEARCH, Protocols & guidelines < HEALTH SERVICES ADMINISTRATION & MANAGEMENT, Dementia < NEUROLOGY, Quality in health care < HEALTH SERVICES ADMINISTRATION & MANAGEMENT, Dementia, Patient-Centered Care

SCHOLARONE™ Manuscripts

For peer review only - http://bmjopen.bmj.com/site/about/guidelines.xhtml

I, the Submitting Author has the right to grant and does grant on behalf of all authors of the Work (as defined in the below author licence), an exclusive licence and/or a non-exclusive licence for contributions from authors who are: i) UK Crown employees; ii) where BMJ has agreed a CC-BY licence shall apply, and/or iii) in accordance with the terms applicable for US Federal Government officers or employees acting as part of their official duties; on a worldwide, perpetual, irrevocable, royalty-free basis to BMJ Publishing Group Ltd ("BMJ") its licensees and where the relevant Journal is co-owned by BMJ to the co-owners of the Journal, to publish the Work in this journal and any other BMJ products and to exploit all rights, as set out in our licence.

The Submitting Author accepts and understands that any supply made under these terms is made by BMJ to the Submitting Author unless you are acting as an employee on behalf of your employer or a postgraduate student of an affiliated institution which is paying any applicable article publishing charge ("APC") for Open Access articles. Where the Submitting Author wishes to make the Work available on an Open Access basis (and intends to pay the relevant APC), the terms of reuse of such Open Access shall be governed by a Creative Commons licence – details of these licences and which Creative Commons licence will apply to this Work are set out in our licence referred to above.

Other than as permitted in any relevant BMJ Author's Self Archiving Policies, I confirm this Work has not been accepted for publication elsewhere, is not being considered for publication elsewhere and does not duplicate material already published. I confirm all authors consent to publication of this Work and authorise the granting of this licence.

For peer review only

TITLE PAGE

Title

Using experience-based co-design (EBCD) to develop high-level design principles for a visual identification system for people with dementia in acute hospital ward settings

Alastair S Macdonald *
The Glasgow School of Art
167 Renfrew Street
Glasgow G3 6RQ, Scotland, UK
a.macdonald@gsa.ac.uk
https://orcid.org/0000-0001-9282-6229
tel: 07811-186-717

Karolina Kuberska
THIS Institute (The Healthcare Improvement Studies Institute)
University of Cambridge
Strangeways Research Laboratory
Cambridge CB1 8RN, England, UK
karolina.kuberska@thisinstitute.cam.ac.uk
https://orcid.org/0000-0002-9610-1863

Naomi Stockley
Point of Care Foundation
The Foundry, 17 Oval Way,
London SE11 5RR, England, UK
naomistockley@pointofcarefoundation.org.uk

Bev Fitzsimons
Point of Care Foundation
The Foundry, 17 Oval Way,
London SE11 5RR, England, UK
BevFitzsimons@pointofcarefoundation.org.uk
https://orcid.org/0000-0001-9749-8877

* Corresponding author

For peer review only

KEYWORDS

Patient identification systems, people with dementia, co-design, EBCD, experience-based co-design, community participation, design principles

WORD COUNT

Excluding title page, abstract, references, figures and tables: 3917

ABSTRACT

Objectives
Using codesign to improve the quality of care is a well-established approach in healthcare improvement. We tested a modified co-design process to develop a set of high-level design principles for visual identification systems (VIS) for hospitalised people with dementia. Routinely used in UK hospitals, these systems help staff quickly recognise and adapt to additional needs. None of the VIS in use in the UK has been rigorously evaluated or been subject to systematic development informed by design principles. We designed and ran remote workshops in three phases with carers of people with dementia and healthcare staff. In phase 1 we presented participants with scenarios based on findings from prior research, prompting participants to discuss their own experiences of VIS. Phase 2 used more future-focused scenarios, prompting participants to co-design improved VIS. In phase 3, a set of provisional design principles developed from our analysis of phases 1 and 2 data were discussed.

Setting
Due to pandemic restrictions, our workshops with carers and healthcare staff were held online.

Participants
A total of 26 carers and 9 staff took part in a pilot and three separate workshops.

Results
We identified a set of six dementia-friendly design principles for improving the effectiveness of VIS. Participants suggested that, in addition to the use of an identifier and key personal information, professional standards training, effective information and records management, and improved means to involve carers and/or families were key to the effective operation of VIS, leading us to expand a narrow understanding of a VIS. Using scenario-led co-design approach can help trigger useful discussions with staff and carer groups, identify current problems with VIS, and develop a set of high-level design principles for their improvement. These principles reveal day-to-day frictions that require further attention and resolution.

STRENGTHS AND LIMITATIONS OF THIS STUDY

- Designing and running a programme of online workshops using scenario-based narratives is feasible and may be a means of reducing some of the time, cost, and accessibility implications of holding in-person workshops.
- Presenting narratives on slide shows with voice-over can effectively replace videoed witness accounts typically used in EBCD studies.
- The EBCD approach can be used in studies focused on abstract, high level design, such as principles for visual identification systems.
- The study would have benefited from a broader representation of healthcare professionals including, e.g., ward managers, health care assistants, out-patients' department staff, and peripatetic staff such as porters.
- Future studies should involve, with appropriate support, patients with dementia and their carers.

For peer review only

INTRODUCTION

People with dementia and some other forms of cognitive impairment in hospital settings face a range of risks that present important challenges for care. One possible intervention to support care is what is commonly referred to as a visual identification system (VIS). This can help staff recognise people with cognitive impairment quickly and easily, and adapt their care accordingly. VIS have normally been understood as having two key elements, an 'identifier' and 'information'. The identifier is usually an easily noticeable sign or symbol, such as a specially coloured wristband, flower or butterfly.(1,2) The presence of the identifier signifies to staff that further important information about the patient's care and personal needs should be consulted and appropriate action taken; this additional information can be made readily available via patient profile documents, e.g., a patient passport,(3) 'This is me' booklet,(4) 'About Me' records,(5) or bedside poster. The Royal College of Psychiatrists recommends that hospitals have a system in place to ensure staff are aware of a patient's cognitive impairment.(6,7) The 2019 report stated that most UK hospitals are using some type of system.(7) However, the College does not recommend a specific form such a system should take, recognising that existing systems are implemented locally and they are likely to vary in cost and effectiveness.(7,8) Although VIS are routinely used within the NHS, some issues have been identified.(9) A number of localised versions of VIS are in use, but none has been rigorously evaluated or subject to systematic development informed by dementia-friendly design principles.

The DA VINCI (Developing A Visual IdeNtification method for people with Cognitive Impairment in institutional settings) programme looked at the use of VIS for people with dementia in hospital settings, with a view to identifying an existing system that might be further developed or, alternatively, co-designing a new system around the needs and preferences of patients, carers and health professionals to ensure acceptability and workability in practice.(8) DA VINCI took a multi-disciplinary approach involving four linked studies from different organisations. These comprised: a survey of current practice around the visual identification of people with cognitive impairment in hospital settings;(6) an analysis of the ethical and legal issues involved in the use of such systems;(10) a qualitative study, including in-depth case studies of and interviews about current applications of VIS in hospitals with staff, people with dementia and their carers;(11) and a co-design study comprising a series of participatory workshops with carers and staff to co-develop a set of design principles. This paper describes the co-design study, the high-level principles identified and how these were produced.

Study question and objective

The earlier DA VINCI survey and qualitative study identified various issues due to the proliferation of systems, suggesting that developing another VIS may be the wrong approach. We decided to explore the possibility of using an established co-design approach to identify a set of high-level principles to guide the development and use of any VIS, both existing and those developed in future. Our objective was to co-develop and evaluate these high-level principles with healthcare professionals and carers of people with dementia.

Co-design

Co-design is a collaborative process in which multiple stakeholders have input. It evolves as a process, maturing and adapting as it takes place. Co-design also involves a transformation of ordinary power relations between stakeholders and aims to generate collective ownership.(12) Experience-based co-design (EBCD) is a structured approach that has been developed to enable 'staff and patients (or other service users) to co-design services and/or care pathways, together in partnership'.(13) EBCD has been used to achieve quality improvements in healthcare settings by bringing staff and patients together through a six-stage (or accelerated five-stage) process structured within a single study.(14,15) Examples include improvements to a mental health inpatient service and pre-treatment care pathway

For peer review only - http://bmjopen.bmj.com/site/about/guidelines.xhtml

for cancer patients.(16,17) Normally, in the early ‘discovery’ phase of EBCD, trigger films (videoed witness accounts of particular issues relating to the service in question) are created to prompt discussion at separate, and then joint, staff and patient events.(18) Staff and patient groups then come together during a later ‘co-design’ stage to suggest improvements to healthcare delivery and experience. We modified the EBCD approach given the separate study set-up in the DA VINCI programme, relying on the findings of the qualitative study(11) for our discovery phase. Also, due to the pandemic, we were unable to create trigger films to prompt discussion. Instead, we developed a set of scenario-based narratives relying on the findings of the qualitative study and prior experiences of our study team. We ran the workshops online.

METHODS

Materials: scenario-based narratives

We prepared a series of text documents describing short scenarios facing a fictional patient with dementia (Alice) admitted to hospital. These highlighted several dementia-related issues particular to Alice at a hospital-, ward-, and patient-level, interspersed with key questions around issues that potentially affected Alice’s care where a VIS might have an important role to play. Two further sets of scenarios were similarly developed, for patient George with Lewy bodies dementia and ward manager Claire. All three were translated into an illustrated slideshow format with added actor voice-overs. We showed some slides with the actor voice-over, paused and asked questions (see Figures 1 and 2, and supplemental file 1) to prompt a discussion about the highlighted issues and then moved on to the next slides and questions. Given the variety of VIS in use in UK hospitals, the scenarios and questions were intentionally generic rather than specific to any single VIS and were designed to support a structured workshop of about two hours’ duration. Workshops were run by BF and NS, professional facilitators experienced in the application of the experience-based co-design approach within healthcare through the Point of Care Foundation (PoCF). AM, a designer working with healthcare professionals using design-led co-development methods, assisted with note taking and some Phase 2 facilitation.

[Insert Figure 1 and caption here]

[Insert Figure 2 and caption here]

Study design

The three scenarios with voice-overs described above were shared with DA VINCI’s Expert Collaborative Group for comment and then piloted in a workshop involving five carers (Table 1). There, participants found it difficult to shift from pre-occupations with their experiences of existing VIS to think more speculatively about improvements. We therefore decided to hold the workshops in two separate phases a week apart. Phase 1 focused on identifying critical issues with the status quo; phase 2 allowed for more speculative thinking to improve these.(19) The phase 1 scenarios covered: i) the frail older patient Alice, her VIS, being in hospital, and issues of consent for the use of the VIS; ii) patient George, and his care while being moved around the hospital; and iii) ward manager Claire, responsible for ensuring staff consulted relevant information and provided person-centred care. The phase 2 scenarios focussed on improving three specific aspects: i) the identifier; ii) the information; and iii) how the VIS could function better. Phase 1 and phase 2 workshops were held with carers and staff separately; all participants were sent a copy of the slides prior to these workshops for their consideration. Following analysis of data from these two phases, we drafted a set of provisional design principles. These were discussed in the phase 3 joint workshop with carers and staff. This reviewed set of principles and feedback were then brought to a

For peer review only

meeting of the Expert Collaborative Group (ECG) for discussion and consolidated after addressing their feedback. Table 1 summarises the study design.

STUDY DESIGN
**Study Design proposal involving the DA VINCI partners and the Expert Collaborative Group**
↓
**Development of workshop materials, piloting the ‘status quo’ scenarios with carers (n=5)**
↓
**Phase 1: separate workshops for staff (n=2) and carers (n=7) using the *‘status quo’* scenarios exploring issues with existing visual information systems**<br>**Phase 2: separate workshops for staff (n=3) and carers (n=7) using the *‘what if?’* scenarios exploring improvements to visual information systems**
↓
**Analysis of data from phase 1 and 2 workshops, leading to a provisional set of design principles discussed with DA VINCI partners**
↓
**Phase 3: joint workshop with staff (n=4) and carers (n=7) exploring the concept of, viability of, and issues with the provisional set of design principles**
↓
**Analysis of feedback from Phase 3 workshop, incorporating recommendations of the Expert Collaborative Group, to confirm our findings**

Table 1. Study design, showing the position of the three workshop phases within the study design, participant numbers in each, the materials used, and nature of the questions explored. Supplemental material 1 provides details of the structure and questions for each of the three workshops.

For peer review only

Recruitment

On behalf of the Point of Care Foundation (PoCF), the charity Dementia Carers Count circulated an invitation to take part in the workshops to their members. Interested participants had to contact the PoCF. There was a range of expertise among the carers who participated: length of time caring; type and severity of the dementia being cared for; setting in which care is/was provided; and experiences of hospital care, and the variability of hospital routines. Healthcare staff were recruited through a call on social media from the PoCF. Everyone who responded was invited to take part. All carers who responded and participated were women. Healthcare staff participating represented a range of expertise including old age psychiatry and specialist dementia nursing; all but one were women. Table 1 provides participant numbers at each stage. All workshop participants were offered compensation for their time in line with the INVOLVE (the UK's public participation charity)

guidelines, which set out recommended rates of reimbursement for public participation in research.(20) For the purposes of this study, all participants (whether staff or carer) were treated as public participants in research and were compensated accordingly.

Consideration of appropriate workshop tools for participants

As we were keen to remove barriers to participation, make the workshops as inclusive and as accessible as possible and so lessen the cognitive load, we decided against the use of any other tools than a proprietary online conferencing tool (Zoom). Many people were familiar with this tool by the time the workshops were held (autumn 2021), whereas many would not be familiar with other remote participative tools, such as collaborative online whiteboards.

Data collection and initial analysis

The qualitative analysis was informed by Ramanadhan et al.'s pragmatic theoretical framework, focusing on obtaining a practical understanding of real-world issues.(21) Their paper offers guidance on pragmatic approaches to analysis, which informed the approach in this study. They define the pragmatic approach as 'strategic combining and borrowing from established qualitative approaches to meet the needs of a given study'.(21) This approach led us to decide explicitly on an inductive process to generate an interpretation primarily grounded in and driven by the data (rather than existing theory). We privileged the perspectives of participants rather than ourselves as researchers and we intentionally connected the analysis plan to the outputs needed i.e. the design principles. Researchers AM, BF and NS gathered field notes during all workshops including, wherever possible, verbatim remarks. Each researcher independently coded their field notes and identified the themes around challenges and benefits of using VIS. These were then compared, and a consensus on the key themes was reached between the researchers in a series of online meetings. All workshops were video recorded, and if there were areas of lack of clarity or agreement about the key themes, the recordings were consulted to help resolve the issue. We analysed our field notes focusing on recurrent themes and patterns of experiences from field notes or paraphrasing common ideas from the conversations that took place in workshops. We describe the process of drafting and refining the co-design principles below.

Patient and Public Involvement

No patients were involved in the design, conduct, reporting, or dissemination plans of this study. Due to the pandemic, workshop participants did not include people with dementia, but any future studies would involve, with appropriate support, people with dementia and their carers as their experience should be key to nuancing the design principles of a VIS. The study teams were assisted by the Expert Collaborative Group (ECG) of the DA VINCI programme composed of healthcare professionals working with patients with dementia, carers for people with dementia, and third sector representatives, who were invited to comment on the methodology and findings of the studies throughout the project. Our initial study plans were shared with the ECG at the outset, the detailed methodology was shared mid-way through the project, ahead of the delivery of the workshops, and the ECG were invited to comment on our first draft design principles. The results of the study have been disseminated to all staff and carer participants, partner study teams and the ECG.

Ethical considerations

The project followed ethical procedures with written information provided in formats suitable for the target participants. The methods and activities undertaken in these workshops were chosen with the needs of people with dementia and their families in mind. Given the restrictions due to the COVID pandemic, workshops were organized and delivered remotely. Facilitation was undertaken by researchers familiar with the co-design approach and with experience of working with individuals who might be vulnerable, due to the personal nature of the subject of discussion. Researchers reassured participants that there were alternative ways to contribute if they were not comfortable using a computer or tablet, for example

For peer review only

offering to send a printed version of workshop materials and asking for comments in whatever way was most comfortable for them. Consent was gained to record the online workshops and to use anonymised quotes, and images of design ideas in our report. No participant was identifiable in any reports to the funder or in other outputs from the study. We checked regularly whether people were happy to continue and emphasised that they could withdraw from the workshops at any time. We also conducted a follow-up evaluation of workshops, so we could understand the experience of people taking part.

RESULTS

We coalesced recurrent subthemes into distinct, identifiable themes, highlighting the key issues. Examples of the key themes identified from the coalescence of points arising from our workshop notes are shown and summarised in Table 2.

THEMES, SUBTHEMES AND KEY POINTS ARISING FROM WORKSHOP ANALYSIS		
**Theme**	**Subtheme**	**Key points identified from workshop notes**
Training in dementia care	Lack of dementia expertise<br><br>Lack of awareness of relevance of the symbol	Experiential training helps staff really understand what it's like to have dementia<br><br>The UK Core Skills Training Framework includes dementia awareness training for every member of staff. Tier 2 training is required for every patient-facing member of staff.<br><br>Training sessions need to be more frequent; many staff members don't have up-to-date training.
The identifier and links to information	Lack of consistency between hospitals<br><br>No link to data	Information about diagnosis needs to link to alert on the Electronic Patient Records (EPR).<br><br>Linking primary care records with hospital records such that if a person with dementia presents to hospital, specialist dementia service is notified.<br><br>Critical information should be presented succinctly (not 4-5 pages).
Information	Information should be relevant, succinct, and easy to access	Include key pieces of information about the person (preferences for name, physical contact, food, Lasting Power of Attorney (LPA), Do Not Resuscitate (DNR), etc.) and ways to contact family and carers.<br><br>Patient profile documents would benefit from a redesign towards presenting key information in a succinct way.
Family participation	Input of family is vital<br><br>Involving family to establish patient preferences and legal provisions.	Routinely contacting family and carers, especially when contact with patient is difficult to avoid making assumptions about legal provisions (e.g. Do Not Resuscitate orders).
Standardisation	Ensuring standard of care	Expectation that patients receive similar levels of care irrespective of which hospital they stay at.

Table 2. Themes, subthemes and key points identified from workshop analysis.

Training

Participants often mentioned the need for regular professional standards training. Of particular concern was high staff turnover and the challenges with ensuring consistency of understanding and communication, including using positive language when discussing patients with dementia, across both regular and bank staff. The need for training also applied to other non-clinical staff, such as porters. Carers emphasised the importance of institutional leadership and organisational culture. They also highlighted that experiential training sessions can be more effective for staff, as healthcare professionals are more likely to retain the information if they are learning by doing. There was consensus that the training needed to be of sufficient quality – recognising that mandatory training, especially online, can be of poor quality.

Linking the identifier to key personal information

Participants pointed out the problems arising from an individual's key personal information not accompanying them around various healthcare settings. Some saw the solution in technology, such as smart-linking key personal information to the identifier (e.g., through a QR or bar code), to make it readily available at each point in the care pathway. Some participants saw value in relevant summary information about the patient also being available in a physical form, suggesting that there may be situations where staff might not have time or opportunity for an extra step of scanning a code or checking the electronic patient record.

Integration and management of records

Participants often mentioned that key medical and personal information often exists in separate electronic and paper-based systems which are not integrated. Participants felt that VIS could be instrumental to much better information integration and management along the whole patient care pathway, to include key search functions, automated alerts and links to that individual's care support needs. Again, many suggested that technology may offer useful solutions, from wearables to individual devices with screens at patient bedside.

Enabling carers and families to provide key information

Participants highlighted the need for up-to-date, readily available, accessible and prioritised key information about each hospitalised individual with dementia, e.g., preferred name, food preferences, allergies, ability to communicate and/or give consent, Lasting Power of Attorney, and Do Not Resuscitate orders, to enable appropriate, personalised care. Relatives and carers felt, where possible, they should be enabled to input and update key personal information and emphasised the importance of being able to connect to their family member via a video call.

Standardising the identifier

Perhaps the most contentious issue to arise relating to the identifier was what participants referred to as "standardisation". Many workshop participants considered that a standardised, nationally mandated identifier, applied on admission with assumed consent (unless revoked), might provide greater consistency in recognising patients with a confirmed diagnosis of dementia across different healthcare organisations, particularly given staff mobility across different sites using different systems. However, workshop participants who were healthcare professionals had concerns about how difficult it would be to implement a unified system across the whole NHS given that different VIS are already in existence.

DESIGN PRINCIPLES

Plsek et al. state that '[d]esign rules are heuristic statements in the form: If you want to achieve outcome Y in situation S, something like X might help.'(22) Our approach to deriving design principles (using the term 'principles' in preference to 'rules') from the Phase 1 and 2

For peer review only

thematic analysis was adapted from their concept, which ‘helps convert the tacit knowledge of organizational change agents into explicit, actionable knowledge’.(22) We used an adapted version of their methods 3 (‘listen to stories of change efforts told by change leaders, operational managers, and front-line staff and then extract design rules off-line [via review of transcripts or notes]) and 4 (‘pose hypothetical scenarios to those experienced in organizational change, ask them to “think aloud” regarding how they would approach the situation, and then extract design rules off-line’) to extract from our findings, through a number of iterations, a set of five draft principles for the joint phase 3 workshop. We provided these in concise form to all participants in a single sheet, which we supplemented with additional explanations at the workshop with further expansion on the principles. We invited participants to comment on individual principles in small breakout groups and at plenary sessions. We asked: ‘If these principles were adopted, would: hospital staff recognise people with dementia more quickly; respond to their needs more readily; and provide better, more personalised care?’ BF, NS and AM took notes of the discussion.

In the phase 3 workshop and in the feedback from the members of the Expert Collaborative Group (ECG), participants generally agreed that some of the principles would be more problematic to implement than others, e.g., improved IT systems where the various clinical records, such as from hospital, GP, care home and memory clinic records, could be better integrated with readily accessible key personal information. The point was also made that relying purely on electronic records was risky and that supplementary paper-based information would be essential. When asked which of the principles would be more fundamentally important to implement, phase 3 participants debated two in particular: training (contingent on organisational culture and leadership) and the form of the identifier (a wristband, symbol etc.).

Opinions were divided over which principle was the most important. Some participants thought that a national, standardised identifier would be relatively easy to implement, especially with third-sector support for dissemination. However, as pointed out by members of the ECG given how well-established some systems already are, a single nationally mandated identifier would be difficult, if not impossible, to implement. With regards to training, inadequately trained staff and lack of an ongoing training programme for all patient-facing employees, particularly for new or agency staff, could lead to them not being able to interpret the identifier in a way that supports patients as individuals. On a number of occasions, training was referred to as the ‘bedrock principle’.

A key point from both the phase 3 workshop and the subsequent ECG discussion highlighted the interdependency of the principles which, if adopted singularly and piecemeal, would be unlikely to achieve the goal of more consistent, personalised care for hospitalised people with dementia. In other words, these principles need to be implemented systemically and as a set, if their benefits are to be realised. As a result, we added the interdependency of principles as a further principle. The feedback we received from phase 3, the ECG, and the project partners helped refine further our principles. The challenge involved making the principles simple yet adaptable so that they can serve a large, varied population in the most effective way possible. We provide a summary of the six working principles we arrived at through the phase 3 workshop in Table 3.

For peer review only - http://bmjopen.bmj.com/site/about/guidelines.xhtml

DESIGN PRINCIPLES
1. The hospital trust provides a professionally trained workforce and an appropriate culture of care.
2. The symbol is easily recognisable and well understood.
3. Key personal information is readily available and accessible.
4. Key personal information is integrated into the Electronic Patient Record (EPR).
5. Relatives and carers are involved in providing key information and monitoring care.
6. The principles need to function as a system to be successful.

Table 3. The set of 6 design principles resulting from the study.

DISCUSSION

Using an experience-based co-design approach

Whereas there are many variations in the design of VIS, we believe this has been the first attempt to develop a set of high-level principles to guide the design and use of VIS, through a participative co-design approach. Our study shows that designing and running a programme of online workshops using scenario-based narratives is feasible and that these may reduce some of the time and cost while improving accessibility in comparison to in-person workshops. Presenting narratives on slide shows with voice-overs can effectively replace videoed witness accounts typically used in experience-based co-design studies. The experience-based co-design approach can also be used in evaluation studies focused on abstract, high-level design, such as developing principles for visual identification systems.

Reconciling abstract principles with practical reality

In phases 1 and 2, we focussed on the more tangible elements of the VIS – the identifier and the key information and how these might be made to work better, which in turn provoked responses about issues concerning the less visible elements, leading to our findings. We acknowledge that formulating our findings into principles using Plsek et al.'s heuristic was challenging for us (requiring several iterations) and that these were not fully resolved in their initial form in the phase 3 workshop. None-the-less, these principles served as a means of engaging participants in a more abstract level of discussion, along the lines of 'If we were to propose a set of design principles such as these, what would be your response be?'. Participants readily engaged with this more abstract form of discussion.

The value of the phase 3 and Expert Collaborative Group feedback to our draft principles was to reveal the 'sticky materiality of practical encounters',(23) i.e. the potential gap between high-level principles and the friction-laden character of situations on the ground, as well as the need for designing around what happens in reality of how identifiers for people with dementia are used in practice. Therefore, the sixth principle, emphasising the need for the first five principles to be implemented simultaneously is crucial. Discussing draft principles also highlighted the importance of a participatory process, whereby a proposed improvement, in prototype form (in this case our draft principles), can be interrogated and iteratively refined.

For peer review only

Broadening the understanding of an effective VIS

While the two elements – the identifier and some form of document holding key personal information – are undeniably important, it became clear that for a VIS to function effectively, other elements – as identified in our findings – play a vital role in a functioning of the system that benefits the patient with dementia. For instance, lack of training may mean that even though a symbol is used for a hospitalised patient with dementia, some staff members may not be able to interpret it as a prompt. Involving carers in providing key information and monitoring care can help ensure that staff are able to adjust their care to the needs of each patient (e.g., using preferred name or assisting with meals) rather than to exclusively rely on their broad training.

Strengths and limitations

Although the numbers of workshop participants were low, they were appropriate for online meetings, offering everyone a chance to share their opinions and expertise. That safe space encouraged developing a bond among the participants despite limited workshop duration, which in turn allowed for insightful and richer discussions and developing consensus.
The study would have benefited from a broader representation of healthcare professionals including, e.g., ward managers, health care assistants, and peripatetic staff such as porters. Future studies should involve patients with dementia and their carers.

CONCLUSION

Our study shows that an adapted co-design approach using scenarios can trigger useful discussions with staff and carer groups, identify problems with VIS, and engage participants in an abstract discussion about high-level design principles; and how these might guide the improvement of VIS.(20) Equally importantly, there was a consensus that the efficiency of the principles relied on their interdependent functioning. If only some principles are adopted singularly and piecemeal, this could lead to inconsistent, less personalised care. Thus, these principles need to be implemented systemically and as a set, if their benefits are to be realised. While we recognise these principles are not yet fully resolved, we have made progress towards better defining these principles. Constraints on the normal experience-based co-design approach imposed by the linked-study commissioning method and the pandemic proved an initial challenge; ultimately, they were not an impediment to creatively developing an effective approach. This method could potentially be applied to other forms of service evaluation and improvement initiatives.

Acknowledgements

We acknowledge the helpful feedback, at all stages in our study from Graham Martin, Director of Research at THIS Institute, and at key points, from our Expert Collaborative Group. Actor Francesca Isherwood provided the voice-overs for the Phase 1 materials. Joanna Goodrich presented the study with NS to the HSRUK conference in Sheffield in July 2022. We gratefully acknowledge the participation of the carers and staff in the workshops, and members of the Expert Collaborative Group for their constructive input. We are grateful to the charity Dementia Carers Count for connecting us with the carers included in this study. The icons in figures 1 and 2 are used by kind permission of Flaticon.com.

Contributor Statement

BF, NS and AM were the researchers, led by BF, comprising the co-design study and all contributed to the drafting of this manuscript, led by AM. KK, reviewed and substantially edited and contributed to the manuscript. All authors reviewed the final version.

For peer review only

Competing Interests

The authors declare no conflict of interest. The funders had no role in the design of the study; in the collection, analyses or interpretation of data; in the writing of the manuscript, or in the decision to publish the results.

Funding

Health Foundation, Grant/Award Number: RHZF/001 - RG88620; Health Services and Delivery Research Programme, Grant/ Award Number: NF-SI-0617-10026

Data Sharing Statement

Not applicable. Hand-written notes only taken for later thematic analysis.

Ethical Approval Statement

As service improvement, this EBCD study did not require an ethics review.

REFERENCES

1. Wye Valley NHS Trust. Forget me not [Internet]. Herefordshire Council; 2018 [cited 2022 Sep 8]. Available from: https://www.wyevalley.nhs.uk/visitors-and-patients/county-hospital-(acute)/in-patients/dementia-care/forget-me-not-1.aspx

2. The Butterfly Scheme. The Butterfly Scheme | Reaching out to people with dementia and to anyone needing memory support [Internet]. 2017 [cited 2022 Sep 8]. Available from: https://butterflyscheme.org.uk/

3. East Sussex Healthcare NHS Trust. This is me care passport [Internet]. 2013 [cited 2022 Sep 8]. Available from: https://www.esht.nhs.uk/wp-content/uploads/2017/10/This-is-me-My-Care-Passport.pdf

4. Alzheimer's Society. This is me [Internet]. 2022 [cited 2022 Sep 8]. Available from: https://www.alzheimers.org.uk/get-support/publications-factsheets/this-is-me

5. Professional Records Standards Body. About me [Internet]. 2022 [cited 2022 Sep 8]. Available from: https://theprsb.org/standards/aboutme/

6. Kuberska K, Dixon-Woods M, Martin G. Visual identifier systems for patients with cognitive impairment in healthcare settings: a survey of practice in UK hospitals. International Journal of Older People Nursing. 2022;

7. Royal College of Psychiatrists. National Audit of Dementia Care in General Hospitals 2018–2019. Round Four Audit Report. London: Royal College of Psychiatrists; 2019 p. 105.

8. THIS Institute. Developing a visual identification method for people with cognitive impairment in institutional settings [Internet]. THIS Institute - The Healthcare Improvement Studies Institute. 2020 [cited 2022 Sep 8]. Available from: https://www.thisinstitute.cam.ac.uk/research-projects/davinci/

9. Featherstone K, Boddington P, Northcott A. Using Signs and Symbols to Label Hospital Patients with a Dementia Diagnosis: Help or Hindrance to Care? Narrative Inquiry in Bioethics. 2020;10(1):49–61.

10. Brigden T, Mitchell C, Ordish J, Hall A. Visual identifiers in the care of people with dementia: An ethical and legal analysis [Internet]. Cambridge: PHG Foundation; 2020

For peer review only

[cited 2022 May 9]. Available from: https://www.phgfoundation.org/media/44/download/visual-identifiers-in-the-care-of-people-with-dementia.pdf?v=1&inline=1

11. Sutton E, Armstrong N, Locock L, Conroy S, Tarrant C. Visual identifiers for people with dementia in hospitals: a qualitative study of a classification system for improving quality of care. BMJ Quality & Safety. forthcoming;

12. Donetto S, Pierri P, Tsianakas V, Robert G. Experience-based Co-design and Healthcare Improvement: Realizing Participatory Design in the Public Sector. The Design Journal. 2015 Jun 1;18(2):227–48.

13. Point of Care Foundation. 1. What is Experience-based co-design? [Internet]. Point of Care Foundation. 2021 [cited 2023 Feb 9]. Available from: https://www.pointofcarefoundation.org.uk/resource/experience-based-co-design-ebcd-toolkit/step-by-step-guide/1-experience-based-co-design/

14. Point of Care Foundation. EBCD: Experience-based co-design toolkit [Internet]. Point of Care Foundation. n.d. [cited 2022 Sep 8]. Available from: https://www.pointofcarefoundation.org.uk/resource/experience-based-co-design-ebcd-toolkit/

15. Goodrich J. Why experience-based co-design improves the patient experience. The Journal of Health Design. 2018 Mar 22;3(1):84–5.

16. Springham N, Robert G. Experience based co-design reduces formal complaints on an acute mental health ward. BMJ Open Quality. 2015 Jan 1;4(1):u209153.w3970.

17. Brady GC, Goodrich J, Roe JWG. Using experience-based co-design to improve the pre-treatment care pathway for people diagnosed with head and neck cancer. Support Care Cancer. 2020 Feb;28(2):739–45.

18. Locock L, Robert G, Boaz A, Vougioukalou S, Shuldham C, Fielden J, et al. Using a national archive of patient experience narratives to promote local patient-centered quality improvement: an ethnographic process evaluation of “accelerated” experience-based co-design. J Health Serv Res Policy. 2014 Oct;19(4):200–7.

19. Dunne A, Raby F. Speculative everything: design, fiction, and social dreaming. Cambridge, Massachusetts ; London: The MIT Press; 2013. 224 p.

20. INVOLVE, NIHR. Policy on payment of fees and expenses for members of the public actively involved with INVOLVE [Internet]. 2016 [cited 2022 Sep 8]. Available from: https://www.invo.org.uk/wp-content/uploads/2016/05/INVOLVE-internal-payment-policy-2016-final-1.pdf

21. Ramanadhan S, Revette AC, Lee RM, Aveling EL. Pragmatic approaches to analyzing qualitative data for implementation science: an introduction. Implement Sci Commun. 2021 Jun 29;2(1):70.

22. Plsek P, Bibby J, Whitby E. Practical Methods for Extracting Explicit Design Rules Grounded in the Experience of Organizational Managers. The Journal of Applied Behavioral Science. 2007 Mar 1;43(1):153–70.

23. Tsing AL. Friction: An Ethnography of Global Connection. Princeton: Princeton University Press; 2005. 336 p.

For peer review only

FIGURE LEGENDS

Figure 1. The introductory slide from the stage 1 workshop to explain the concept of the Visual Identification System with two key elements (within the blue circle) and how these relate to other relevant aspects of the hospital care environment.

Figure 2. A further slide from the stage 1 workshop illustrating key components of the Visual Information System for a fictional patient 'Alice', i.e., the 'visual identifier' and the 'information element'.

For peer review only

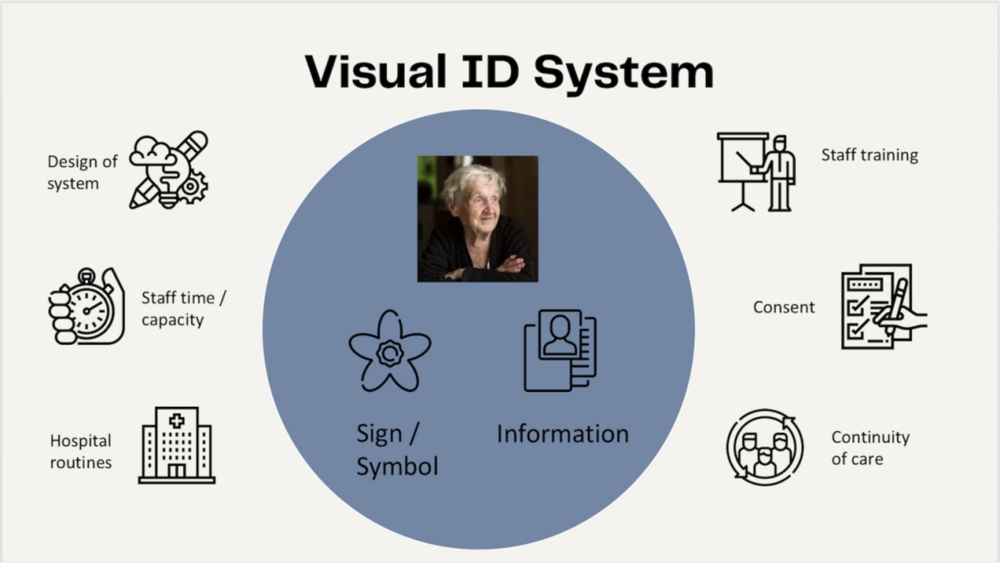

Figure 1. The introductory slide from the stage 1 workshop to explain the concept of the Visual Identification System with two key elements (within the blue circle) and how these relate to other relevant aspects of the hospital care environment.

119x67mm (300 x 300 DPI)

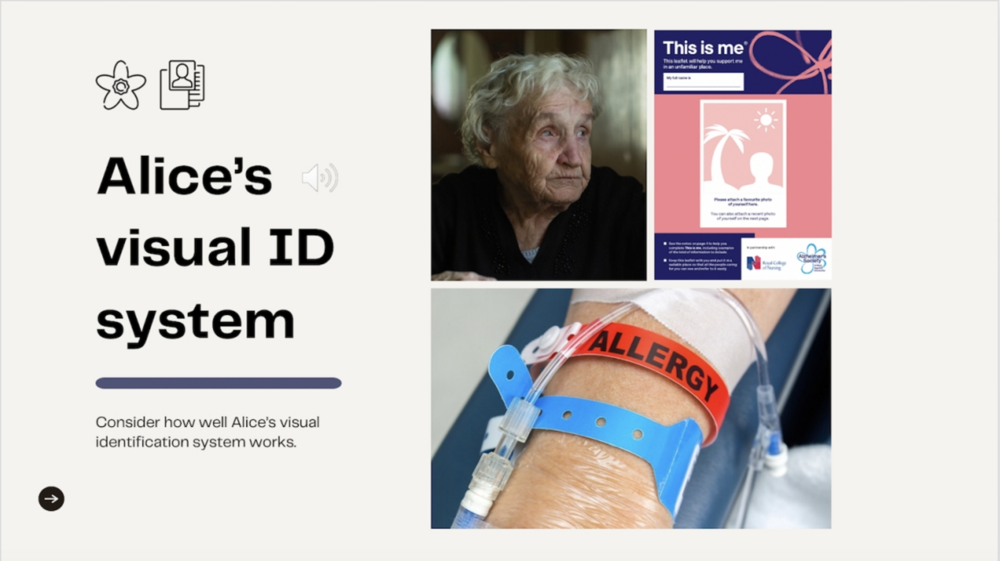

Figure 2. A further slide from the stage 1 workshop illustrating key components of the Visual Information System for a fictional patient 'Alice', i.e., the 'visual identifier' and the 'information element'.

119x67mm (300 x 300 DPI)

PHASE 1 DISCOVERY WORKSHOP	
**Titles of scenario slides**	**Content and questions delivered via actor voice-over**
**Introduction**	Explanation of Visual Identification Systems (VIS)
**Alice's Story**<br>**Alice's VIS**<br>**Alice's hospital**	*What information about Alice is needed to:*<br>• *keep her safe while she is in hospital?*<br>• *help staff communicate well with her?*<br>• *make sure she gets the right treatment and care?*<br>• *avoid agitation and distress?*<br>• *make sure she is treated like a person?*
**Consenting to the identifier**<br>**Issues of Consent**	• *Should patients/relatives give consent for an identifier to be used? If so, what should the care team do to get consent?*<br>• *What do patients/relatives need to know about the implications of having an identifier?*
**George's story**<br>**George's visual IDs**<br>**Continuity of care**	• *How can we make sure that it is clear to all hospital staff what the identifier means, and what they should do when they see it?*<br>• *How can we ensure essential information is easy to find and that all staff know about and consult the information?*
**Claire's story**<br>**Claire's hospital**<br>**Visual identifiers**	• *Given their busy schedules, how can we ensure staff have the time to consult the patient information documents?*<br>• *How can we better ensure that appropriate care continues from one shift to the next?*
**What did we miss?**	• *What did we miss?*

For peer review only

PHASE 2 CO-DESIGN WORKSHOP	
**Scenario slides**	**Content and questions delivered verbally by researchers**
**Recap/intro: VIS**<br>**How it should work**<br><br>**The sign/symbol element**<br>**The information element**<br><br>**How do we overcome these obstacles?**	
**6 hat thinking**	
**The sign / symbol**	• *What should the symbol tell you about the individual?*<br>• *Do different systems across different sites lead to problems?*<br>• *What might make it better?*
	• *What if the symbol was the wristband (perhaps coloured), was personalized and smart in some way - like a unique RIFD, tracking, QR or barcode?*<br>• *What would be the advantages of this approach?*<br>• *What would make this work well?*
**The information (1)**	• *Could a standardised tool be used as part of the admissions process?*<br>• *How best might this information be gathered?*
**The information (2)**	• *What is the key information that is needed to allow them to be treated both as a person and for their specific dementia and other care needs?*<br>• *Would it be best if the essential information about the patient is stored and managed as part of the EPR system, rather than separately stored information?*
**Making the system work (1)**	• *Who would have responsibility for completing this?*<br>• *How could we make sure it is absolutely clear whose responsibility it is to enter the information and that it gets done?*<br>• *How can we make sure that all staff know about, can find, consult and act on this information?*
**Making the system work (2)**	• *Assuming the details are on the system, how would it benefit the bedside care of people with dementia?*<br>• *How can we make this system work when the patient is moved away from the bedside?*
**What did we miss?**	• *What did we miss?*

For peer review only

PHASE 3 VERIFICATION WORKSHOP	
**Stage**	**Content and questions delivered verbally by researchers**
**Welcome and introductions (Plenary)**	
**Recap on progress made in prior workshops (Plenary)**	
**Introduction to the design principles (Plenary)**	• What MUST happen, or MUST be in place, for the system to work so that:<br>1. hospital staff recognise people with dementia more quickly<br>2. respond to their needs more readily<br>3. and provide better, more personalised care
**Sense-checking the design principles (Plenary)**	• Do you have any questions about the principles?<br>• What did you think of the idea of a set of five co-dependent principles?
**Critiquing the design principles (Small mixed groups)**	• Which principle(s) do you most strongly agree with? Why?<br>• Are there any principles on the list that you wouldn't necessarily include? Why?<br>• Are there any missing from the list?
**Feedback (Plenary)**	
**How to implement the design principles (Small mixed groups)**	• How viable is it to implement any/some/all of these principles?<br>• If so, what would need to happen first?<br>• What would help implement them?<br>• What would get in the way?
**Feedback (Plenary)**	
**Anything we missed? (Plenary)**	• *What did we miss?*

For peer review only

BMJ Open

Using experience-based co-design (EBCD) to develop high-level design principles for a visual identification system for people with dementia in acute hospital ward settings

Journal:	*BMJ Open*
Manuscript ID	bmjopen-2022-069352.R2
Article Type:	Original research
Date Submitted by the Author:	06-Apr-2023
Complete List of Authors:	Macdonald, Alastair; The Glasgow School of Art, School of Design<br>Kuberska, Karolina; University of Cambridge, THIS Institute (The Healthcare Improvement Studies Institute), Department of Public Health and Primary Care<br>Stockley, Naomi; The Point of Care Foundation<br>Fitzsimons, Bev; The Point of Care Foundation
**Primary Subject Heading**:	Qualitative research
Secondary Subject Heading:	Health services research, Patient-centred medicine
Keywords:	QUALITATIVE RESEARCH, Protocols & guidelines < HEALTH SERVICES ADMINISTRATION & MANAGEMENT, Dementia < NEUROLOGY, Quality in health care < HEALTH SERVICES ADMINISTRATION & MANAGEMENT, Dementia, Patient-Centered Care

SCHOLARONE™
Manuscripts

I, the Submitting Author has the right to grant and does grant on behalf of all authors of the Work (as defined in the below author licence), an exclusive licence and/or a non-exclusive licence for contributions from authors who are: i) UK Crown employees; ii) where BMJ has agreed a CC-BY licence shall apply, and/or iii) in accordance with the terms applicable for US Federal Government officers or employees acting as part of their official duties; on a worldwide, perpetual, irrevocable, royalty-free basis to BMJ Publishing Group Ltd ("BMJ") its licensees and where the relevant Journal is co-owned by BMJ to the co-owners of the Journal, to publish the Work in this journal and any other BMJ products and to exploit all rights, as set out in our licence.

The Submitting Author accepts and understands that any supply made under these terms is made by BMJ to the Submitting Author unless you are acting as an employee on behalf of your employer or a postgraduate student of an affiliated institution which is paying any applicable article publishing charge ("APC") for Open Access articles. Where the Submitting Author wishes to make the Work available on an Open Access basis (and intends to pay the relevant APC), the terms of reuse of such Open Access shall be governed by a Creative Commons licence – details of these licences and which Creative Commons licence will apply to this Work are set out in our licence referred to above.

Other than as permitted in any relevant BMJ Author's Self Archiving Policies, I confirm this Work has not been accepted for publication elsewhere, is not being considered for publication elsewhere and does not duplicate material already published. I confirm all authors consent to publication of this Work and authorise the granting of this licence.

For peer review only

TITLE PAGE

Title

Using experience-based co-design (EBCD) to develop high-level design principles for a visual identification system for people with dementia in acute hospital ward settings

Alastair S Macdonald *
The Glasgow School of Art
167 Renfrew Street
Glasgow G3 6RQ, Scotland, UK
a.macdonald@gsa.ac.uk
https://orcid.org/0000-0001-9282-6229
tel: 07811-186-717

Karolina Kuberska
THIS Institute (The Healthcare Improvement Studies Institute)
University of Cambridge
Strangeways Research Laboratory
Cambridge CB1 8RN, England, UK
karolina.kuberska@thisinstitute.cam.ac.uk
https://orcid.org/0000-0002-9610-1863

Naomi Stockley
Point of Care Foundation
The Foundry, 17 Oval Way,
London SE11 5RR, England, UK
naomistockley@pointofcarefoundation.org.uk

Bev Fitzsimons
Point of Care Foundation
The Foundry, 17 Oval Way,
London SE11 5RR, England, UK
BevFitzsimons@pointofcarefoundation.org.uk
https://orcid.org/0000-0001-9749-8877

* Corresponding author

KEYWORDS

Patient identification systems, people with dementia, co-design, EBCD, experience-based co-design, community participation, design principles

WORD COUNT

Excluding title page, abstract, references, figures and tables: 4039

For peer review only

ABSTRACT

Objectives

Using codesign to improve the quality of care is a well-established approach in healthcare improvement. We tested a modified co-design process to develop a set of high-level design principles for visual identification systems (VIS) for hospitalised people with dementia. Routinely used in UK hospitals, these systems help staff quickly recognise and adapt to additional needs. None of the VIS in use in the UK has been rigorously evaluated or been subject to systematic development informed by design principles. We designed and ran remote workshops in three phases with carers of people with dementia and healthcare staff. In phase 1 we presented participants with scenarios based on findings from prior research, prompting participants to discuss their own experiences of VIS. Phase 2 used more future-focused scenarios, prompting participants to co-design improved VIS. In phase 3, a set of provisional design principles developed from our analysis of phases 1 and 2 data were discussed.

Setting

Due to pandemic restrictions, our workshops with carers and healthcare staff were held online.

Participants

A total of 26 carers and 9 staff took part in a pilot and three separate workshops.

Results

We identified a set of six dementia-friendly design principles for improving the effectiveness of VIS. Participants suggested that, in addition to the use of an identifier and key personal information, professional standards training, effective information and records management, and improved means to involve carers and/or families were key to the effective operation of VIS, leading us to expand a narrow understanding of a VIS. Using scenario-led co-design approach can help trigger useful discussions with staff and carer groups, identify current problems with VIS, and develop a set of high-level design principles for their improvement. These principles reveal day-to-day frictions that require further attention and resolution.

STRENGTHS AND LIMITATIONS OF THIS STUDY

- The EBCD approach is adaptable and can be used to promote discussion of abstract high-level service principles as well as identify practical improvements to service design and delivery.
- Designing and running a programme of online workshops using scenario-based narratives is a feasible and accessible alternative to holding in-person workshops.
- The EBCD structure helps bonding between researchers and participants, in turn allowing for insightful and richer discussions, and for developing consensus.
- The study would have benefited from a broader representation of healthcare professionals including, e.g., ward managers, health care assistants, out-patients' department staff, and peripatetic staff such as porters, as well as people with dementia and their carers.

For peer review only

INTRODUCTION

People with dementia and some other forms of cognitive impairment in hospital settings face a range of risks that present important challenges for care. One possible intervention to support care is what is commonly referred to as a visual identification system (VIS). This can help staff recognise people with cognitive impairment quickly and easily, and adapt their care accordingly. VIS have normally been understood as having two key elements, an 'identifier' and 'information'. The identifier is usually an easily noticeable sign or symbol, such as a specially coloured wristband, flower or butterfly.(1,2) The presence of the identifier signifies to staff that further important information about the patient's care and personal needs should be consulted and appropriate action taken; this additional information can be made readily available via patient profile documents, e.g., a patient passport,(3) 'This is me' booklet,(4) 'About Me' records,(5) or bedside poster. The Royal College of Psychiatrists recommends that hospitals have a system in place to ensure staff are aware of a patient's cognitive impairment.(6,7) The 2019 report stated that most UK hospitals are using some type of system.(7) However, the College does not recommend a specific form such a system should take, recognising that existing systems are implemented locally and they are likely to vary in cost and effectiveness.(7,8) Although VIS are routinely used within the NHS, some issues have been identified.(9) A number of localised versions of VIS are in use, but none has been rigorously evaluated or subject to systematic development informed by dementia-friendly design principles.

The DA VINCI (Developing A Visual IdeNtification method for people with Cognitive Impairment in institutional settings) programme looked at the use of VIS for people with dementia in hospital settings, with a view to identifying an existing system that might be further developed or, alternatively, co-designing a new system around the needs and preferences of patients, carers and health professionals to ensure acceptability and workability in practice.(8) DA VINCI took a multi-disciplinary approach involving four linked studies from different organisations. These comprised: a survey of current practice around the visual identification of people with cognitive impairment in hospital settings;(6) an analysis of the ethical and legal issues involved in the use of such systems;(10) a qualitative study, including in-depth case studies of and interviews about current applications of VIS in hospitals with staff, people with dementia and their carers;(11) and a co-design study comprising a series of participatory workshops with carers and staff to co-develop a set of design principles. This paper describes the co-design study, the high-level principles identified and how these were produced.

Study question and objective

The earlier DA VINCI survey and qualitative study identified various issues due to the proliferation of systems, suggesting that developing another VIS may be the wrong approach. We decided to explore the possibility of using an established co-design approach to identify a set of high-level principles to guide the development and use of any VIS, both existing and those developed in future. Our objective was to co-develop and evaluate these high-level principles with healthcare professionals and carers of people with dementia.

Co-design

Co-design is a collaborative process in which multiple stakeholders have input. It evolves as a process, maturing and adapting as it takes place. Co-design also involves a transformation of ordinary power relations between stakeholders and aims to generate collective ownership.(12) Experience-based co-design (EBCD) is a structured approach that has been developed to enable 'staff and patients (or other service users) to co-design services and/or care pathways, together in partnership'.(13) EBCD has been used to achieve quality improvements in healthcare settings by bringing staff and patients together through a six-stage (or accelerated five-stage) process structured within a single study.(14,15) Examples include improvements to a mental health inpatient service and pre-treatment care pathway

For peer review only

for cancer patients.(16,17) Normally, in the early ‘discovery’ phase of EBCD, trigger films (videoed witness accounts of particular issues relating to the service in question) are created to prompt discussion at separate, and then joint, staff and patient events.(18) Staff and patient groups then come together during a later ‘co-design’ stage to suggest improvements to healthcare delivery and experience. We modified the EBCD approach given the separate study set-up in the DA VINCI programme, relying on the findings of the qualitative study(11) for our discovery phase. Also, due to the pandemic, we were unable to create trigger films to prompt discussion. Instead, we developed a set of scenario-based narratives relying on the findings of the qualitative study and prior experiences of our study team. We ran the workshops online.

METHODS

Materials: scenario-based narratives

We prepared a series of text documents describing short scenarios facing a fictional patient with dementia (Alice) admitted to hospital. These highlighted several dementia-related issues particular to Alice at a hospital-, ward-, and patient-level, interspersed with key questions around issues that potentially affected Alice’s care where a VIS might have an important role to play. Two further sets of scenarios were similarly developed, for patient George with Lewy bodies dementia and ward manager Claire. All three were translated into an illustrated slideshow format with added actor voice-overs. We showed some slides with the actor voice-over, paused and asked questions (see Figures 1 and 2, and supplemental file 1) to prompt a discussion about the highlighted issues and then moved on to the next slides and questions. Given the variety of VIS in use in UK hospitals, the scenarios and questions were intentionally generic rather than specific to any single VIS and were designed to support a structured workshop of about two hours’ duration. Workshops were run by BF and NS, professional facilitators experienced in the application of the experience-based co-design approach within healthcare through the Point of Care Foundation (PoCF). AM, a designer working with healthcare professionals using design-led co-development methods, assisted with note taking and some Phase 2 facilitation.

[Insert Figure 1 and caption here]

[Insert Figure 2 and caption here]

Study design

The three scenarios with voice-overs described above were shared with DA VINCI’s Expert Collaborative Group for comment and then piloted in a workshop involving five carers (Table 1). There, participants found it difficult to shift from pre-occupations with their experiences of existing VIS to think more speculatively about improvements. We therefore decided to hold the workshops in two separate phases a week apart. Phase 1 focused on identifying critical issues with the status quo; phase 2 allowed for more speculative thinking to improve these.(19) The phase 1 scenarios covered: i) the frail older patient Alice, her VIS, being in hospital, and issues of consent for the use of the VIS; ii) patient George, and his care while being moved around the hospital; and iii) ward manager Claire, responsible for ensuring staff consulted relevant information and provided person-centred care. The phase 2 scenarios focussed on improving three specific aspects: i) the identifier; ii) the information; and iii) how the VIS could function better. Phase 1 and phase 2 workshops were held with carers and staff separately; all participants were sent a copy of the slides prior to these workshops for their consideration. Following analysis of data from these two phases, we drafted a set of provisional design principles. These were discussed in the phase 3 joint workshop with carers and staff. This reviewed set of principles and feedback were then brought to a

For peer review only

meeting of the Expert Collaborative Group (ECG) for discussion and consolidated after addressing their feedback. Table 1 summarises the study design.

STUDY DESIGN
**Study Design proposal involving the DA VINCI partners and the Expert Collaborative Group**
**Development of workshop materials, piloting the 'status quo' scenarios with carers (n=5)**
**Phase 1: separate workshops for staff (n=2) and carers (n=7) using the *'status quo'* scenarios exploring issues with existing visual information systems**<br>**Phase 2: separate workshops for staff (n=3) and carers (n=7) using the *'what if?'* scenarios exploring improvements to visual information systems**
**Analysis of data from phase 1 and 2 workshops, leading to a provisional set of design principles discussed with DA VINCI partners**
**Phase 3: joint workshop with staff (n=4) and carers (n=7) exploring the concept of, viability of, and issues with the provisional set of design principles**
**Analysis of feedback from Phase 3 workshop, incorporating recommendations of the Expert Collaborative Group, to confirm our findings**

Table 1. Study design, showing the position of the three workshop phases within the study design, participant numbers in each, the materials used, and nature of the questions explored. Supplemental material 1 provides details of the structure and questions for each of the three workshops.

For peer review only

Recruitment

On behalf of the Point of Care Foundation (PoCF), the charity Dementia Carers Count circulated an invitation to take part in the workshops to their members. Interested participants had to contact the PoCF. There was a range of expertise among the carers who participated: length of time caring; type and severity of the dementia being cared for; setting in which care is/was provided; and experiences of hospital care, and the variability of hospital routines. Healthcare staff were recruited through a call on social media from the PoCF. Everyone who responded was invited to take part. All carers who responded and participated were women. Healthcare staff participating represented a range of expertise including old age psychiatry and specialist dementia nursing; all but one were women. Table 1 provides participant numbers at each stage. All workshop participants were offered compensation for their time in line with the INVOLVE (the UK's public participation charity)

guidelines, which set out recommended rates of reimbursement for public participation in research.(20) For the purposes of this study, all participants (whether staff or carer) were treated as public participants in research and were compensated accordingly.

Consideration of appropriate workshop tools for participants

As we were keen to remove barriers to participation, make the workshops as inclusive and as accessible as possible and so lessen the cognitive load, we decided against the use of any other tools than a proprietary online conferencing tool (Zoom). Many people were familiar with this tool by the time the workshops were held (autumn 2021), whereas many would not be familiar with other remote participative tools, such as collaborative online whiteboards.

Data collection and initial analysis

The qualitative analysis was informed by Ramanadhan et al.'s pragmatic theoretical framework, focusing on obtaining a practical understanding of real-world issues.(21) Their paper offers guidance on pragmatic approaches to analysis, which informed the approach in this study. They define the pragmatic approach as 'strategic combining and borrowing from established qualitative approaches to meet the needs of a given study'.(21) This approach led us to decide explicitly on an inductive process to generate an interpretation primarily grounded in and driven by the data (rather than existing theory). We privileged the perspectives of participants rather than ourselves as researchers and we intentionally connected the analysis plan to the outputs needed i.e. the design principles. Researchers AM, BF and NS gathered field notes during all workshops including, wherever possible, verbatim remarks. Each researcher independently coded their field notes and identified the themes around challenges and benefits of using VIS. These were then compared, and a consensus on the key themes was reached between the researchers in a series of online meetings. All workshops were video recorded, and if there were areas of lack of clarity or agreement about the key themes, the recordings were consulted to help resolve the issue. We analysed our field notes focusing on recurrent themes and patterns of experiences from field notes or paraphrasing common ideas from the conversations that took place in workshops. We describe the process of drafting and refining the co-design principles below.

Patient and Public Involvement

No patients were involved in the design, conduct, reporting, or dissemination plans of this study. Due to the pandemic, workshop participants did not include people with dementia, but any future studies would involve, with appropriate support, people with dementia and their carers as their experience should be key to nuancing the design principles of a VIS. The study teams were assisted by the Expert Collaborative Group (ECG) of the DA VINCI programme composed of healthcare professionals working with patients with dementia, carers for people with dementia, and third sector representatives, who were invited to comment on the methodology and findings of the studies throughout the project. Our initial study plans were shared with the ECG at the outset, the detailed methodology was shared mid-way through the project, ahead of the delivery of the workshops, and the ECG were invited to comment on our first draft design principles. The results of the study have been disseminated to all staff and carer participants, partner study teams and the ECG.

Ethical considerations

The project followed ethical procedures with written information provided in formats suitable for the target participants. The methods and activities undertaken in these workshops were chosen with the needs of people with dementia and their families in mind. Given the restrictions due to the COVID pandemic, workshops were organized and delivered remotely. Facilitation was undertaken by researchers familiar with the co-design approach and with experience of working with individuals who might be vulnerable, due to the personal nature of the subject of discussion. Researchers reassured participants that there were alternative ways to contribute if they were not comfortable using a computer or tablet, for example

For peer review only

offering to send a printed version of workshop materials and asking for comments in whatever way was most comfortable for them. Consent was gained to record the online workshops and to use anonymised quotes, and images of design ideas in our report. No participant was identifiable in any reports to the funder or in other outputs from the study. We checked regularly whether people were happy to continue and emphasised that they could withdraw from the workshops at any time. We also conducted a follow-up evaluation of workshops, so we could understand the experience of people taking part.

RESULTS

We coalesced recurrent subthemes into distinct, identifiable themes, highlighting the key issues. Examples of the key themes identified from the coalescence of points arising from our workshop notes are shown and summarised in Table 2.

THEMES, SUBTHEMES AND KEY POINTS ARISING FROM WORKSHOP ANALYSIS		
**Theme**	**Subtheme**	**Key points identified from workshop notes**
Training in dementia care	Lack of dementia expertise<br><br>Lack of awareness of relevance of the symbol	Experiential training helps staff really understand what it's like to have dementia<br><br>The UK Core Skills Training Framework includes dementia awareness training for every member of staff. Tier 2 training is required for every patient-facing member of staff.<br><br>Training sessions need to be more frequent; many staff members don't have up-to-date training.
The identifier and links to information	Lack of consistency between hospitals<br><br>No link to data	Information about diagnosis needs to link to alert on the Electronic Patient Records (EPR).<br><br>Linking primary care records with hospital records such that if a person with dementia presents to hospital, specialist dementia service is notified.<br><br>Critical information should be presented succinctly (not 4-5 pages).
Information	Information should be relevant, succinct, and easy to access	Include key pieces of information about the person (preferences for name, physical contact, food, Lasting Power of Attorney (LPA), Do Not Resuscitate (DNR), etc.) and ways to contact family and carers.<br><br>Patient profile documents would benefit from a redesign towards presenting key information in a succinct way.
Family participation	Input of family is vital<br><br>Involving family to establish patient preferences and legal provisions.	Routinely contacting family and carers, especially when contact with patient is difficult to avoid making assumptions about legal provisions (e.g. Do Not Resuscitate orders).
Standardisation	Ensuring standard of care	Expectation that patients receive similar levels of care irrespective of which hospital they stay at.

Table 2. Themes, subthemes and key points identified from workshop analysis.

Training

Participants often mentioned the need for regular professional standards training. Of particular concern was high staff turnover and the challenges with ensuring consistency of understanding and communication, including using positive language when discussing patients with dementia, across both regular and bank staff. The need for training also applied to other non-clinical staff, such as porters. Carers emphasised the importance of institutional leadership and organisational culture. They also highlighted that experiential training sessions can be more effective for staff, as healthcare professionals are more likely to retain the information if they are learning by doing. There was consensus that the training needed to be of sufficient quality – recognising that mandatory training, especially online, can be of poor quality.

Linking the identifier to key personal information

Participants pointed out the problems arising from an individual's key personal information not accompanying them around various healthcare settings. Some saw the solution in technology, such as smart-linking key personal information to the identifier (e.g., through a QR or bar code), to make it readily available at each point in the care pathway. Some participants saw value in relevant summary information about the patient also being available in a physical form, suggesting that there may be situations where staff might not have time or opportunity for an extra step of scanning a code or checking the electronic patient record.

Integration and management of records

Participants often mentioned that key medical and personal information often exists in separate electronic and paper-based systems which are not integrated. Participants felt that VIS could be instrumental to much better information integration and management along the whole patient care pathway, to include key search functions, automated alerts and links to that individual's care support needs. Again, many suggested that technology may offer useful solutions, from wearables to individual devices with screens at patient bedside.

Enabling carers and families to provide key information

Participants highlighted the need for up-to-date, readily available, accessible and prioritised key information about each hospitalised individual with dementia, e.g., preferred name, food preferences, allergies, ability to communicate and/or give consent, Lasting Power of Attorney, and Do Not Resuscitate orders, to enable appropriate, personalised care. Relatives and carers felt, where possible, they should be enabled to input and update key personal information and emphasised the importance of being able to connect to their family member via a video call.

Standardising the identifier

Perhaps the most contentious issue to arise relating to the identifier was what participants referred to as "standardisation". Many workshop participants considered that a standardised, nationally mandated identifier, applied on admission with assumed consent (unless revoked), might provide greater consistency in recognising patients with a confirmed diagnosis of dementia across different healthcare organisations, particularly given staff mobility across different sites using different systems. However, workshop participants who were healthcare professionals had concerns about how difficult it would be to implement a unified system across the whole NHS given that different VIS are already in existence.

DESIGN PRINCIPLES

Plsek et al. state that '[d]esign rules are heuristic statements in the form: If you want to achieve outcome Y in situation S, something like X might help.'(22) Our approach to deriving design principles (using the term 'principles' in preference to 'rules') from the Phase 1 and 2

For peer review only

thematic analysis was adapted from their concept, which ‘helps convert the tacit knowledge of organizational change agents into explicit, actionable knowledge’.(22) We used an adapted version of their methods 3 (‘listen to stories of change efforts told by change leaders, operational managers, and front-line staff and then extract design rules off-line [via review of transcripts or notes]) and 4 (‘pose hypothetical scenarios to those experienced in organizational change, ask them to “think aloud” regarding how they would approach the situation, and then extract design rules off-line’) to extract from our findings, through a number of iterations, a set of five draft principles for the joint phase 3 workshop. We provided these in concise form to all participants in a single sheet, which we supplemented with additional explanations at the workshop with further expansion on the principles. We invited participants to comment on individual principles in small breakout groups and at plenary sessions. We asked: ‘If these principles were adopted, would: hospital staff recognise people with dementia more quickly; respond to their needs more readily; and provide better, more personalised care?’ BF, NS and AM took notes of the discussion.

In the phase 3 workshop and in the feedback from the members of the Expert Collaborative Group (ECG), participants generally agreed that some of the principles would be more problematic to implement than others, e.g., improved IT systems where the various clinical records, such as from hospital, GP, care home and memory clinic records, could be better integrated with readily accessible key personal information. The point was also made that relying purely on electronic records was risky and that supplementary paper-based information would be essential. When asked which of the principles would be more fundamentally important to implement, phase 3 participants debated two in particular: training (contingent on organisational culture and leadership) and the form of the identifier (a wristband, symbol etc.).

Opinions were divided over which principle was the most important. Some participants thought that a national, standardised identifier would be relatively easy to implement, especially with third-sector support for dissemination. However, as pointed out by members of the ECG given how well-established some systems already are, a single nationally mandated identifier would be difficult, if not impossible, to implement. With regards to training, inadequately trained staff and lack of an ongoing training programme for all patient-facing employees, particularly for new or agency staff, could lead to them not being able to interpret the identifier in a way that supports patients as individuals. On a number of occasions, training was referred to as the ‘bedrock principle’.

A key point from both the phase 3 workshop and the subsequent ECG discussion highlighted the interdependency of the principles which, if adopted singularly and piecemeal, would be unlikely to achieve the goal of more consistent, personalised care for hospitalised people with dementia. In other words, these principles need to be implemented systemically and as a set, if their benefits are to be realised. As a result, we added the interdependency of principles as a further principle. The feedback we received from phase 3, the ECG, and the project partners helped refine further our principles. The challenge involved making the principles simple yet adaptable so that they can serve a large, varied population in the most effective way possible. We provide a summary of the six working principles we arrived at through the phase 3 workshop in Table 3.

For peer review only

DESIGN PRINCIPLES
**1. The hospital trust provides a professionally trained workforce and an appropriate culture of care.**
**2. The symbol is easily recognisable and well understood.**
**3. Key personal information is readily available and accessible.**
**4. Key personal information is integrated into the Electronic Patient Record (EPR).**
**5. Relatives and carers are involved in providing key information and monitoring care.**
**6. The principles need to function as a system to be successful.**

Table 3. The set of 6 design principles resulting from the study.

DISCUSSION

Using an experience-based co-design approach

Whereas there are many variations in the design of VIS, we believe this has been the first attempt to develop a set of high-level principles to guide the design and use of VIS, through a participative co-design approach. Our study shows that designing and running a programme of online workshops using scenario-based narratives is feasible and that these may reduce some of the time and cost while improving accessibility in comparison to in-person workshops. Presenting narratives on slide shows with voice-overs can effectively replace videoed witness accounts typically used in experience-based co-design studies. The experience-based co-design approach can also be used in evaluation studies focused on abstract, high-level design, such as developing principles for visual identification systems.

Reconciling abstract principles with practical reality

In phases 1 and 2, we focussed on the more tangible elements of the VIS – the identifier and the key information and how these might be made to work better, which in turn provoked responses about issues concerning the less visible elements, leading to our findings. We acknowledge that formulating our findings into principles using Plsek et al.'s heuristic was challenging for us (requiring several iterations) and that these were not fully resolved in their initial form in the phase 3 workshop. None-the-less, these principles served as a means of engaging participants in a more abstract level of discussion, along the lines of 'If we were to propose a set of design principles such as these, what would be your response be?'. Participants readily engaged with this more abstract form of discussion.

The value of the phase 3 and Expert Collaborative Group feedback to our draft principles was to reveal the 'sticky materiality of practical encounters',(23) i.e. the potential gap between high-level principles and the friction-laden character of situations on the ground, as well as the need for designing around what happens in reality of how identifiers for people with dementia are used in practice. Therefore, the sixth principle, emphasising the need for the first five principles to be implemented simultaneously is crucial. Discussing draft principles also highlighted the importance of a participatory process, whereby a proposed improvement, in prototype form (in this case our draft principles), can be interrogated and iteratively refined.

For peer review only

Broadening the understanding of an effective VIS

While the two elements – the identifier and some form of document holding key personal information – are undeniably important, it became clear that for a VIS to function effectively, other elements – as identified in our findings – play a vital role in a functioning of the system that benefits the patient with dementia. For instance, lack of training may mean that even though a symbol is used for a hospitalised patient with dementia, some staff members may not be able to interpret it as a prompt. Involving carers in providing key information and monitoring care can help ensure that staff are able to adjust their care to the needs of each patient (e.g., using preferred name or assisting with meals) rather than to exclusively rely on their broad training.

Strengths and limitations

The EBCD approach is adaptable and can be used to promote discussion of abstract high-level service principles as well as identify practical improvements to service design and delivery. Designing and running a programme of online workshops using scenario-based narratives is a feasible and accessible alternative to holding in-person workshops.
The EBCD structure helps bonding between researchers and participants, in turn allowing for insightful and richer discussions, and for developing consensus. The study would have benefited from a broader representation of healthcare professionals including, e.g., ward managers, health care assistants, out-patients' department staff, and peripatetic staff such as porters, as well as people with dementia and their carers.

CONCLUSION

Our study shows that an adapted co-design approach using scenarios can trigger useful discussions with staff and carer groups, identify problems with VIS, and engage participants in an abstract discussion about high-level design principles; and how these might guide the improvement of VIS.(20) Equally importantly, there was a consensus that the efficiency of the principles relied on their interdependent functioning. If only some principles are adopted singularly and piecemeal, this could lead to inconsistent, less personalised care. Thus, these principles need to be implemented systemically and as a set, if their benefits are to be realised. While we recognise these principles are not yet fully resolved, we have made progress towards better defining these principles. Constraints on the normal experience-based co-design approach imposed by the linked-study commissioning method and the pandemic proved an initial challenge; ultimately, they were not an impediment to creatively developing an effective approach. This method could potentially be applied to other forms of service evaluation and improvement initiatives.

Acknowledgements

We acknowledge the helpful feedback, at all stages in our study from Graham Martin, Director of Research at THIS Institute, and at key points, from our Expert Collaborative Group. Actor Francesca Isherwood provided the voice-overs for the Phase 1 materials. Joanna Goodrich presented the study with NS to the HSRUK conference in Sheffield in July 2022. We gratefully acknowledge the participation of the carers and staff in the workshops, and members of the Expert Collaborative Group for their constructive input. We are grateful to the charity Dementia Carers Count for connecting us with the carers included in this study. The icons in figures 1 and 2 are used by kind permission of Flaticon.com.

Contributor Statement

BF, NS and AM were the researchers, led by BF, comprising the co-design study and all contributed to the drafting of this manuscript, led by AM. KK, reviewed and substantially edited and contributed to the manuscript. All authors reviewed the final version.

For peer review only

Competing Interests
The authors declare no conflict of interest. The funders had no role in the design of the study; in the collection, analyses or interpretation of data; in the writing of the manuscript, or in the decision to publish the results.

Funding
Health Foundation, Grant/Award Number: RHZF/001 - RG88620; Health Services and Delivery Research Programme, Grant/ Award Number: NF-SI-0617-10026

Data Sharing Statement
Not applicable. Hand-written notes only taken for later thematic analysis.

Ethical Approval Statement
As service improvement, this EBCD study did not require an ethics review.

For peer review only

REFERENCES

1. Wye Valley NHS Trust. Forget me not [Internet]. Herefordshire Council; 2018 [cited 2022 Sep 8]. Available from: https://www.wyevalley.nhs.uk/visitors-and-patients/county-hospital-(acute)/in-patients/dementia-care/forget-me-not-1.aspx

2. The Butterfly Scheme. The Butterfly Scheme | Reaching out to people with dementia and to anyone needing memory support [Internet]. 2017 [cited 2022 Sep 8]. Available from: https://butterflyscheme.org.uk/

3. East Sussex Healthcare NHS Trust. This is me care passport [Internet]. 2013 [cited 2022 Sep 8]. Available from: https://www.esht.nhs.uk/wp-content/uploads/2017/10/This-is-me-My-Care-Passport.pdf

4. Alzheimer's Society. This is me [Internet]. 2022 [cited 2022 Sep 8]. Available from: https://www.alzheimers.org.uk/get-support/publications-factsheets/this-is-me

5. Professional Records Standards Body. About me [Internet]. 2022 [cited 2022 Sep 8]. Available from: https://theprsb.org/standards/aboutme/

6. Kuberska K, Dixon-Woods M, Martin G. Visual identifier systems for patients with cognitive impairment in healthcare settings: a survey of practice in UK hospitals. International Journal of Older People Nursing. 2022;

7. Royal College of Psychiatrists. National Audit of Dementia Care in General Hospitals 2018–2019. Round Four Audit Report. London: Royal College of Psychiatrists; 2019 p. 105.

8. THIS Institute. Developing a visual identification method for people with cognitive impairment in institutional settings [Internet]. THIS Institute - The Healthcare Improvement Studies Institute. 2020 [cited 2022 Sep 8]. Available from: https://www.thisinstitute.cam.ac.uk/research-projects/davinci/

9. Featherstone K, Boddington P, Northcott A. Using Signs and Symbols to Label Hospital Patients with a Dementia Diagnosis: Help or Hindrance to Care? Narrative Inquiry in Bioethics. 2020;10(1):49–61.

10. Brigden T, Mitchell C, Ordish J, Hall A. Visual identifiers in the care of people with dementia: An ethical and legal analysis [Internet]. Cambridge: PHG Foundation; 2020

[cited 2022 May 9]. Available from: https://www.phgfoundation.org/media/44/download/visual-identifiers-in-the-care-of-people-with-dementia.pdf?v=1&inline=1

11. Sutton E, Armstrong N, Locock L, Conroy S, Tarrant C. Visual identifiers for people with dementia in hospitals: a qualitative study of a classification system for improving quality of care. BMJ Quality & Safety. forthcoming;

12. Donetto S, Pierri P, Tsianakas V, Robert G. Experience-based Co-design and Healthcare Improvement: Realizing Participatory Design in the Public Sector. The Design Journal. 2015 Jun 1;18(2):227–48.

13. Point of Care Foundation. 1. What is Experience-based co-design? [Internet]. Point of Care Foundation. 2021 [cited 2023 Feb 9]. Available from: https://www.pointofcarefoundation.org.uk/resource/experience-based-co-design-ebcd-toolkit/step-by-step-guide/1-experience-based-co-design/

14. Point of Care Foundation. EBCD: Experience-based co-design toolkit [Internet]. Point of Care Foundation. n.d. [cited 2022 Sep 8]. Available from: https://www.pointofcarefoundation.org.uk/resource/experience-based-co-design-ebcd-toolkit/

15. Goodrich J. Why experience-based co-design improves the patient experience. The Journal of Health Design. 2018 Mar 22;3(1):84–5.

16. Springham N, Robert G. Experience based co-design reduces formal complaints on an acute mental health ward. BMJ Open Quality. 2015 Jan 1;4(1):u209153.w3970.

17. Brady GC, Goodrich J, Roe JWG. Using experience-based co-design to improve the pre-treatment care pathway for people diagnosed with head and neck cancer. Support Care Cancer. 2020 Feb;28(2):739–45.

18. Locock L, Robert G, Boaz A, Vougioukalou S, Shuldham C, Fielden J, et al. Using a national archive of patient experience narratives to promote local patient-centered quality improvement: an ethnographic process evaluation of “accelerated” experience-based co-design. J Health Serv Res Policy. 2014 Oct;19(4):200–7.

19. Dunne A, Raby F. Speculative everything: design, fiction, and social dreaming. Cambridge, Massachusetts ; London: The MIT Press; 2013. 224 p.

20. INVOLVE, NIHR. Policy on payment of fees and expenses for members of the public actively involved with INVOLVE [Internet]. 2016 [cited 2022 Sep 8]. Available from: https://www.invo.org.uk/wp-content/uploads/2016/05/INVOLVE-internal-payment-policy-2016-final-1.pdf

21. Ramanadhan S, Revette AC, Lee RM, Aveling EL. Pragmatic approaches to analyzing qualitative data for implementation science: an introduction. Implement Sci Commun. 2021 Jun 29;2(1):70.

22. Plsek P, Bibby J, Whitby E. Practical Methods for Extracting Explicit Design Rules Grounded in the Experience of Organizational Managers. The Journal of Applied Behavioral Science. 2007 Mar 1;43(1):153–70.

23. Tsing AL. Friction: An Ethnography of Global Connection. Princeton: Princeton University Press; 2005. 336 p.

For peer review only

FIGURE LEGENDS

Figure 1. The introductory slide from the stage 1 workshop to explain the concept of the Visual Identification System with two key elements (within the blue circle) and how these relate to other relevant aspects of the hospital care environment.

Figure 2. A further slide from the stage 1 workshop illustrating key components of the Visual Information System for a fictional patient 'Alice', i.e., the 'visual identifier' and the 'information element'.

For peer review only

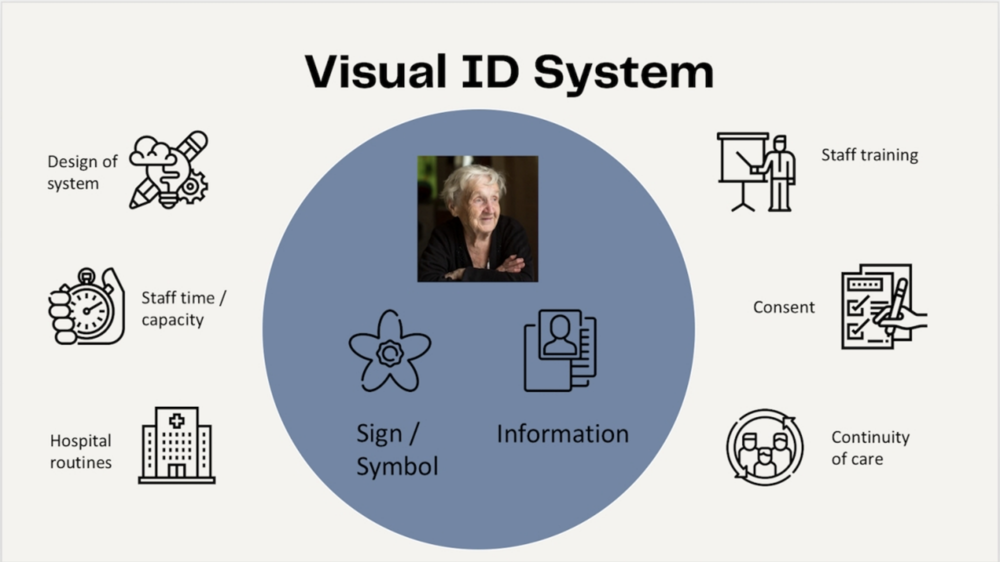

Figure 1. The introductory slide from the stage 1 workshop to explain the concept of the Visual Identification System with two key elements (within the blue circle) and how these relate to other relevant aspects of the hospital care environment.

119x67mm (300 x 300 DPI)

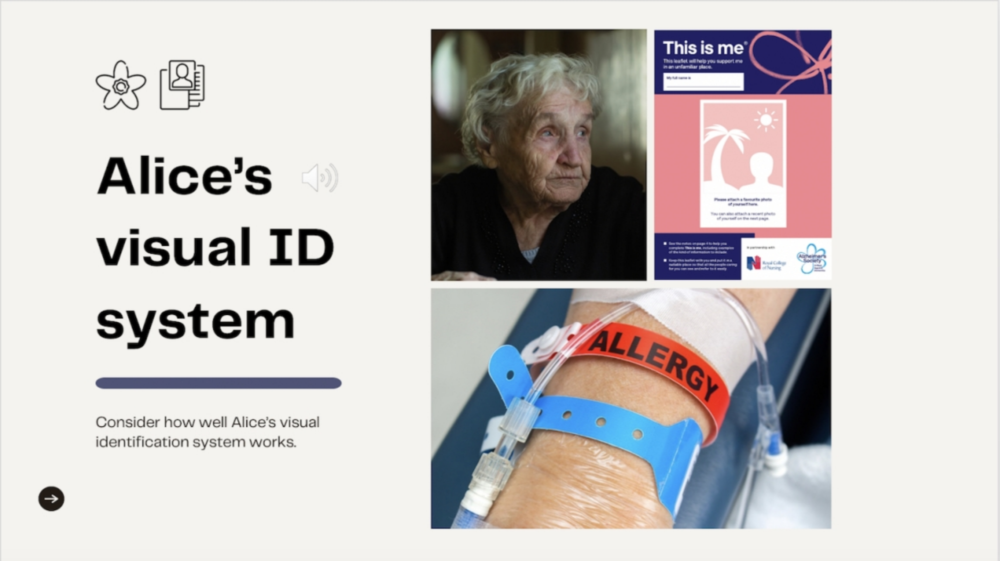

Figure 2. A further slide from the stage 1 workshop illustrating key components of the Visual Information System for a fictional patient 'Alice', i.e., the 'visual identifier' and the 'information element'.

119x67mm (300 x 300 DPI)

PHASE 1 DISCOVERY WORKSHOP	
**Titles of scenario slides**	**Content and questions delivered via actor voice-over**
**Introduction**	Explanation of Visual Identification Systems (VIS)
**Alice's Story**<br>**Alice's VIS**<br>**Alice's hospital**	*What information about Alice is needed to:*<br>• *keep her safe while she is in hospital?*<br>• *help staff communicate well with her?*<br>• *make sure she gets the right treatment and care?*<br>• *avoid agitation and distress?*<br>• *make sure she is treated like a person?*
**Consenting to the identifier**<br>**Issues of Consent**	• *Should patients/relatives give consent for an identifier to be used? If so, what should the care team do to get consent?*<br>• *What do patients/relatives need to know about the implications of having an identifier?*
**George's story**<br>**George's visual IDs**<br>**Continuity of care**	• *How can we make sure that it is clear to all hospital staff what the identifier means, and what they should do when they see it?*<br>• *How can we ensure essential information is easy to find and that all staff know about and consult the information?*
**Claire's story**<br>**Claire's hospital**<br>**Visual identifiers**	• *Given their busy schedules, how can we ensure staff have the time to consult the patient information documents?*<br>• *How can we better ensure that appropriate care continues from one shift to the next?*
**What did we miss?**	• *What did we miss?*

For peer review only

PHASE 2 CO-DESIGN WORKSHOP	
**Scenario slides**	**Content and questions delivered verbally by researchers**
**Recap/intro: VIS**<br>**How it should work**<br><br>**The sign/symbol element**<br>**The information element**<br><br>**How do we overcome these obstacles?**	
**6 hat thinking**	
**The sign / symbol**	• *What should the symbol tell you about the individual?*<br>• *Do different systems across different sites lead to problems?*<br>• *What might make it better?*
	• *What if the symbol was the wristband (perhaps coloured), was personalized and smart in some way - like a unique RIFD, tracking, QR or barcode?*<br>• *What would be the advantages of this approach?*<br>• *What would make this work well?*
**The information (1)**	• *Could a standardised tool be used as part of the admissions process?*<br>• *How best might this information be gathered?*
**The information (2)**	• *What is the key information that is needed to allow them to be treated both as a person and for their specific dementia and other care needs?*<br>• *Would it be best if the essential information about the patient is stored and managed as part of the EPR system, rather than separately stored information?*
**Making the system work (1)**	• *Who would have responsibility for completing this?*<br>• *How could we make sure it is absolutely clear whose responsibility it is to enter the information and that it gets done?*<br>• *How can we make sure that all staff know about, can find, consult and act on this information?*
**Making the system work (2)**	• *Assuming the details are on the system, how would it benefit the bedside care of people with dementia?*<br>• *How can we make this system work when the patient is moved away from the bedside?*
**What did we miss?**	• *What did we miss?*

For peer review only

PHASE 3 VERIFICATION WORKSHOP	
**Stage**	**Content and questions delivered verbally by researchers**
**Welcome and introductions (Plenary)**	
**Recap on progress made in prior workshops (Plenary)**	
**Introduction to the design principles (Plenary)**	• What MUST happen, or MUST be in place, for the system to work so that:<br>1. hospital staff recognise people with dementia more quickly<br>2. respond to their needs more readily<br>3. and provide better, more personalised care
**Sense-checking the design principles (Plenary)**	• Do you have any questions about the principles?<br>• What did you think of the idea of a set of five co-dependent principles?
**Critiquing the design principles (Small mixed groups)**	• Which principle(s) do you most strongly agree with? Why?<br>• Are there any principles on the list that you wouldn't necessarily include? Why?<br>• Are there any missing from the list?
**Feedback (Plenary)**	
**How to implement the design principles (Small mixed groups)**	• How viable is it to implement any/some/all of these principles?<br>• If so, what would need to happen first?<br>• What would help implement them?<br>• What would get in the way?
**Feedback (Plenary)**	
**Anything we missed? (Plenary)**	• *What did we miss?*

For peer review only

BMJ Open

Using experience-based co-design (EBCD) to develop high-level design principles for a visual identification system for people with dementia in acute hospital ward settings

Journal:	*BMJ Open*
Manuscript ID	bmjopen-2022-069352.R3
Article Type:	Original research
Date Submitted by the Author:	14-Apr-2023
Complete List of Authors:	Macdonald, Alastair; The Glasgow School of Art, School of Design<br>Kuberska, Karolina; University of Cambridge, THIS Institute (The Healthcare Improvement Studies Institute), Department of Public Health and Primary Care<br>Stockley, Naomi; The Point of Care Foundation<br>Fitzsimons, Bev; The Point of Care Foundation
**Primary Subject Heading**:	Qualitative research
Secondary Subject Heading:	Health services research, Patient-centred medicine
Keywords:	QUALITATIVE RESEARCH, Protocols & guidelines < HEALTH SERVICES ADMINISTRATION & MANAGEMENT, Dementia < NEUROLOGY, Quality in health care < HEALTH SERVICES ADMINISTRATION & MANAGEMENT, Dementia, Patient-Centered Care

SCHOLARONE™
Manuscripts

I, the Submitting Author has the right to grant and does grant on behalf of all authors of the Work (as defined in the below author licence), an exclusive licence and/or a non-exclusive licence for contributions from authors who are: i) UK Crown employees; ii) where BMJ has agreed a CC-BY licence shall apply, and/or iii) in accordance with the terms applicable for US Federal Government officers or employees acting as part of their official duties; on a worldwide, perpetual, irrevocable, royalty-free basis to BMJ Publishing Group Ltd ("BMJ") its licensees and where the relevant Journal is co-owned by BMJ to the co-owners of the Journal, to publish the Work in this journal and any other BMJ products and to exploit all rights, as set out in our licence.

The Submitting Author accepts and understands that any supply made under these terms is made by BMJ to the Submitting Author unless you are acting as an employee on behalf of your employer or a postgraduate student of an affiliated institution which is paying any applicable article publishing charge ("APC") for Open Access articles. Where the Submitting Author wishes to make the Work available on an Open Access basis (and intends to pay the relevant APC), the terms of reuse of such Open Access shall be governed by a Creative Commons licence – details of these licences and which Creative Commons licence will apply to this Work are set out in our licence referred to above.

Other than as permitted in any relevant BMJ Author's Self Archiving Policies, I confirm this Work has not been accepted for publication elsewhere, is not being considered for publication elsewhere and does not duplicate material already published. I confirm all authors consent to publication of this Work and authorise the granting of this licence.

For peer review only

TITLE PAGE

Title

Using experience-based co-design (EBCD) to develop high-level design principles for a visual identification system for people with dementia in acute hospital ward settings

Alastair S Macdonald *
The Glasgow School of Art
167 Renfrew Street
Glasgow G3 6RQ, Scotland, UK
a.macdonald@gsa.ac.uk
https://orcid.org/0000-0001-9282-6229
tel: 07811-186-717

Karolina Kuberska
THIS Institute (The Healthcare Improvement Studies Institute)
University of Cambridge
Strangeways Research Laboratory
Cambridge CB1 8RN, England, UK
karolina.kuberska@thisinstitute.cam.ac.uk
https://orcid.org/0000-0002-9610-1863

Naomi Stockley
Point of Care Foundation
The Foundry, 17 Oval Way,
London SE11 5RR, England, UK
naomistockley@pointofcarefoundation.org.uk

Bev Fitzsimons
Point of Care Foundation
The Foundry, 17 Oval Way,
London SE11 5RR, England, UK
BevFitzsimons@pointofcarefoundation.org.uk
https://orcid.org/0000-0001-9749-8877

* Corresponding author

KEYWORDS

Patient identification systems, people with dementia, co-design, EBCD, experience-based co-design, community participation, design principles

WORD COUNT

Excluding title page, abstract, references, figures and tables: 4039

For peer review only

ABSTRACT

Objectives

We tested a modified co-design process to develop a set of high-level design principles for visual identification systems (VIS) for hospitalised people with dementia.

Design

We designed and ran remote workshops in three phases with carers of people with dementia and healthcare staff. In phase 1 we presented participants with scenarios based on findings from prior research, prompting participants to discuss their own experiences of VIS. Phase 2 used more future-focused scenarios, prompting participants to co-design improved VIS. In phase 3, a set of provisional design principles developed from our analysis of phases 1 and 2 data were discussed.

Setting

Online workshops.

Participants

A total of 26 carers and 9 healthcare staff took part in a pilot and three separate workshops.

Results

We identified a set of six dementia-friendly design principles for improving the effectiveness of VIS: 1) The hospital trust provides a professionally-trained workforce and an appropriate culture of care; 2) The symbol is easily recognisable and well understood; 3) Key personal information is readily available and accessible; 4) Key personal information is integrated into the Electronic Patient Record; 5) Relatives and carers are involved in providing key information and monitoring care; 6) The principles need to function as a system to be successful. Participants suggested that, in addition to the use of an identifier and key personal information, professional standards training, effective information and records management, and improved means to involve carers and/or families were key to the effective operation of VIS, leading us to expand a narrow understanding of a VIS.

Conclusion

Using scenario-led co-design approach can help trigger useful discussions with staff and carer groups, identify current problems with VIS, and develop a set of high-level design principles for their improvement. These principles reveal day-to-day frictions that require further attention and resolution.

STRENGTHS AND LIMITATIONS OF THIS STUDY

- The EBCD approach is adaptable and can be used to promote discussion of abstract high-level service principles as well as identify practical improvements to service design and delivery.
- Designing and running a programme of online workshops using scenario-based narratives is a feasible and accessible alternative to holding in-person workshops.
- The EBCD structure helps bonding between researchers and participants, in turn allowing for insightful and richer discussions, and for developing consensus.
- The study would have benefited from a broader representation of healthcare professionals including, e.g., ward managers, health care assistants, out-patients' department staff, and peripatetic staff such as porters, as well as people with dementia and their carers.

For peer review only

INTRODUCTION

People with dementia and some other forms of cognitive impairment in hospital settings face a range of risks that present important challenges for care. One possible intervention to support care is what is commonly referred to as a visual identification system (VIS). This can help staff recognise people with cognitive impairment quickly and easily, and adapt their care accordingly. VIS have normally been understood as having two key elements, an 'identifier' and 'information'. The identifier is usually an easily noticeable sign or symbol, such as a specially coloured wristband, flower or butterfly.(1,2) The presence of the identifier signifies to staff that further important information about the patient's care and personal needs should be consulted and appropriate action taken; this additional information can be made readily available via patient profile documents, e.g., a patient passport,(3) 'This is me' booklet,(4) 'About Me' records,(5) or bedside poster. The Royal College of Psychiatrists recommends that hospitals have a system in place to ensure staff are aware of a patient's cognitive impairment.(6,7) The 2019 report stated that most UK hospitals are using some type of system.(7) However, the College does not recommend a specific form such a system should take, recognising that existing systems are implemented locally and they are likely to vary in cost and effectiveness.(7,8) Although VIS are routinely used within the NHS, some issues have been identified.(9) A number of localised versions of VIS are in use, but none has been rigorously evaluated or subject to systematic development informed by dementia-friendly design principles.

The DA VINCI (Developing A Visual IdeNtification method for people with Cognitive Impairment in institutional settings) programme looked at the use of VIS for people with dementia in hospital settings, with a view to identifying an existing system that might be further developed or, alternatively, co-designing a new system around the needs and preferences of patients, carers and health professionals to ensure acceptability and workability in practice.(8) DA VINCI took a multi-disciplinary approach involving four linked studies from different organisations. These comprised: a survey of current practice around the visual identification of people with cognitive impairment in hospital settings;(6) an analysis of the ethical and legal issues involved in the use of such systems;(10) a qualitative study, including in-depth case studies of and interviews about current applications of VIS in hospitals with staff, people with dementia and their carers;(11) and a co-design study comprising a series of participatory workshops with carers and staff to co-develop a set of design principles. This paper describes the co-design study, the high-level principles identified and how these were produced.

Study question and objective

The earlier DA VINCI survey and qualitative study identified various issues due to the proliferation of systems, suggesting that developing another VIS may be the wrong approach. We decided to explore the possibility of using an established co-design approach to identify a set of high-level principles to guide the development and use of any VIS, both existing and those developed in future. Our objective was to co-develop and evaluate these high-level principles with healthcare professionals and carers of people with dementia.

Co-design

Co-design is a collaborative process in which multiple stakeholders have input. It evolves as a process, maturing and adapting as it takes place. Co-design also involves a transformation of ordinary power relations between stakeholders and aims to generate collective ownership.(12) Experience-based co-design (EBCD) is a structured approach that has been developed to enable 'staff and patients (or other service users) to co-design services and/or care pathways, together in partnership'.(13) EBCD has been used to achieve quality improvements in healthcare settings by bringing staff and patients together through a six-stage (or accelerated five-stage) process structured within a single study.(14,15) Examples include improvements to a mental health inpatient service and pre-treatment care pathway

For peer review only

for cancer patients.(16,17) Normally, in the early 'discovery' phase of EBCD, trigger films (videoed witness accounts of particular issues relating to the service in question) are created to prompt discussion at separate, and then joint, staff and patient events.(18) Staff and patient groups then come together during a later 'co-design' stage to suggest improvements to healthcare delivery and experience. We modified the EBCD approach given the separate study set-up in the DA VINCI programme, relying on the findings of the qualitative study(11) for our discovery phase. Also, due to the pandemic, we were unable to create trigger films to prompt discussion. Instead, we developed a set of scenario-based narratives relying on the findings of the qualitative study and prior experiences of our study team. We ran the workshops online.

METHODS

Materials: scenario-based narratives

We prepared a series of text documents describing short scenarios facing a fictional patient with dementia (Alice) admitted to hospital. These highlighted several dementia-related issues particular to Alice at a hospital-, ward-, and patient-level, interspersed with key questions around issues that potentially affected Alice's care where a VIS might have an important role to play. Two further sets of scenarios were similarly developed, for patient George with Lewy bodies dementia and ward manager Claire. All three were translated into an illustrated slideshow format with added actor voice-overs. We showed some slides with the actor voice-over, paused and asked questions (see Figures 1 and 2, and supplemental file 1) to prompt a discussion about the highlighted issues and then moved on to the next slides and questions. Given the variety of VIS in use in UK hospitals, the scenarios and questions were intentionally generic rather than specific to any single VIS and were designed to support a structured workshop of about two hours' duration. Workshops were run by BF and NS, professional facilitators experienced in the application of the experience-based co-design approach within healthcare through the Point of Care Foundation (PoCF). AM, a designer working with healthcare professionals using design-led co-development methods, assisted with note taking and some Phase 2 facilitation.

[Insert Figure 1 and caption here]

[Insert Figure 2 and caption here]

Study design

The three scenarios with voice-overs described above were shared with DA VINCI's Expert Collaborative Group for comment and then piloted in a workshop involving five carers (Table 1). There, participants found it difficult to shift from pre-occupations with their experiences of existing VIS to think more speculatively about improvements. We therefore decided to hold the workshops in two separate phases a week apart. Phase 1 focused on identifying critical issues with the status quo; phase 2 allowed for more speculative thinking to improve these.(19) The phase 1 scenarios covered: i) the frail older patient Alice, her VIS, being in hospital, and issues of consent for the use of the VIS; ii) patient George, and his care while being moved around the hospital; and iii) ward manager Claire, responsible for ensuring staff consulted relevant information and provided person-centred care. The phase 2 scenarios focussed on improving three specific aspects: i) the identifier; ii) the information; and iii) how the VIS could function better. Phase 1 and phase 2 workshops were held with carers and staff separately; all participants were sent a copy of the slides prior to these workshops for their consideration. Following analysis of data from these two phases, we drafted a set of provisional design principles. These were discussed in the phase 3 joint workshop with carers and staff. This reviewed set of principles and feedback were then brought to a

For peer review only

meeting of the Expert Collaborative Group (ECG) for discussion and consolidated after addressing their feedback. Table 1 summarises the study design.

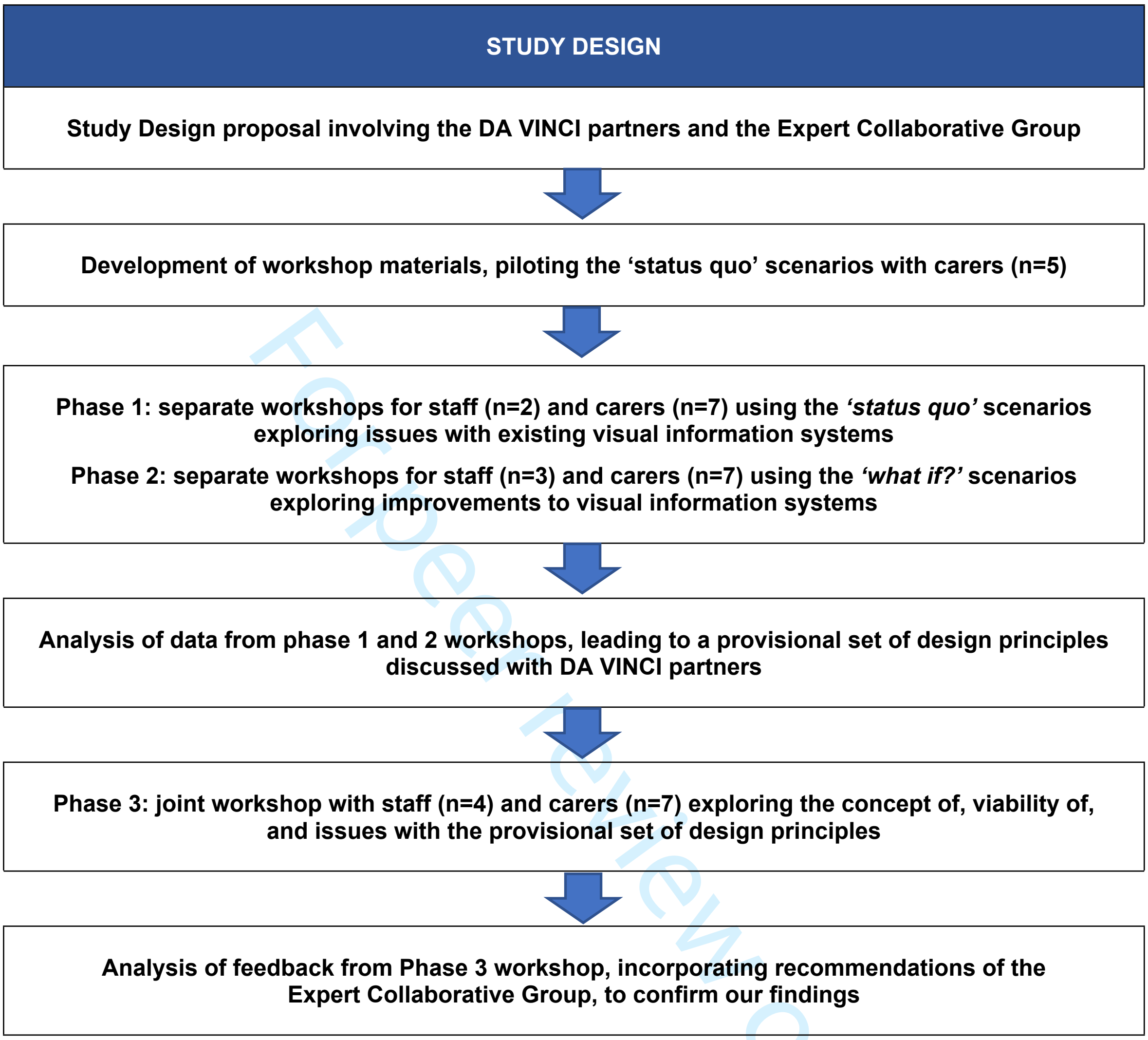

Table 1. Study design, showing the position of the three workshop phases within the study design, participant numbers in each, the materials used, and nature of the questions explored. Supplemental material 1 provides details of the structure and questions for each of the three workshops.

For peer review only

Recruitment

On behalf of the Point of Care Foundation (PoCF), the charity Dementia Carers Count circulated an invitation to take part in the workshops to their members. Interested participants had to contact the PoCF. There was a range of expertise among the carers who participated: length of time caring; type and severity of the dementia being cared for; setting in which care is/was provided; and experiences of hospital care, and the variability of hospital routines. Healthcare staff were recruited through a call on social media from the PoCF. Everyone who responded was invited to take part. All carers who responded and participated were women. Healthcare staff participating represented a range of expertise including old age psychiatry and specialist dementia nursing; all but one were women. Table 1 provides participant numbers at each stage. All workshop participants were offered compensation for their time in line with the INVOLVE (the UK's public participation charity)

guidelines, which set out recommended rates of reimbursement for public participation in research.(20) For the purposes of this study, all participants (whether staff or carer) were treated as public participants in research and were compensated accordingly.

Consideration of appropriate workshop tools for participants

As we were keen to remove barriers to participation, make the workshops as inclusive and as accessible as possible and so lessen the cognitive load, we decided against the use of any other tools than a proprietary online conferencing tool (Zoom). Many people were familiar with this tool by the time the workshops were held (autumn 2021), whereas many would not be familiar with other remote participative tools, such as collaborative online whiteboards.

Data collection and initial analysis

The qualitative analysis was informed by Ramanadhan et al.'s pragmatic theoretical framework, focusing on obtaining a practical understanding of real-world issues.(21) Their paper offers guidance on pragmatic approaches to analysis, which informed the approach in this study. They define the pragmatic approach as 'strategic combining and borrowing from established qualitative approaches to meet the needs of a given study'.(21) This approach led us to decide explicitly on an inductive process to generate an interpretation primarily grounded in and driven by the data (rather than existing theory). We privileged the perspectives of participants rather than ourselves as researchers and we intentionally connected the analysis plan to the outputs needed i.e. the design principles. Researchers AM, BF and NS gathered field notes during all workshops including, wherever possible, verbatim remarks. Each researcher independently coded their field notes and identified the themes around challenges and benefits of using VIS. These were then compared, and a consensus on the key themes was reached between the researchers in a series of online meetings. All workshops were video recorded, and if there were areas of lack of clarity or agreement about the key themes, the recordings were consulted to help resolve the issue. We analysed our field notes focusing on recurrent themes and patterns of experiences from field notes or paraphrasing common ideas from the conversations that took place in workshops. We describe the process of drafting and refining the co-design principles below.

Patient and Public Involvement

No patients were involved in the design, conduct, reporting, or dissemination plans of this study. Due to the pandemic, workshop participants did not include people with dementia, but any future studies would involve, with appropriate support, people with dementia and their carers as their experience should be key to nuancing the design principles of a VIS. The study teams were assisted by the Expert Collaborative Group (ECG) of the DA VINCI programme composed of healthcare professionals working with patients with dementia, carers for people with dementia, and third sector representatives, who were invited to comment on the methodology and findings of the studies throughout the project. Our initial study plans were shared with the ECG at the outset, the detailed methodology was shared mid-way through the project, ahead of the delivery of the workshops, and the ECG were invited to comment on our first draft design principles. The results of the study have been disseminated to all staff and carer participants, partner study teams and the ECG.

Ethical considerations

The project followed ethical procedures with written information provided in formats suitable for the target participants. The methods and activities undertaken in these workshops were chosen with the needs of people with dementia and their families in mind. Given the restrictions due to the COVID pandemic, workshops were organized and delivered remotely. Facilitation was undertaken by researchers familiar with the co-design approach and with experience of working with individuals who might be vulnerable, due to the personal nature of the subject of discussion. Researchers reassured participants that there were alternative ways to contribute if they were not comfortable using a computer or tablet, for example

For peer review only

offering to send a printed version of workshop materials and asking for comments in whatever way was most comfortable for them. Consent was gained to record the online workshops and to use anonymised quotes, and images of design ideas in our report. No participant was identifiable in any reports to the funder or in other outputs from the study. We checked regularly whether people were happy to continue and emphasised that they could withdraw from the workshops at any time. We also conducted a follow-up evaluation of workshops, so we could understand the experience of people taking part.

RESULTS

We coalesced recurrent subthemes into distinct, identifiable themes, highlighting the key issues. Examples of the key themes identified from the coalescence of points arising from our workshop notes are shown and summarised in Table 2.

THEMES, SUBTHEMES AND KEY POINTS ARISING FROM WORKSHOP ANALYSIS		
**Theme**	**Subtheme**	**Key points identified from workshop notes**
Training in dementia care	Lack of dementia expertise<br><br>Lack of awareness of relevance of the symbol	Experiential training helps staff really understand what it's like to have dementia<br><br>The UK Core Skills Training Framework includes dementia awareness training for every member of staff. Tier 2 training is required for every patient-facing member of staff.<br><br>Training sessions need to be more frequent; many staff members don't have up-to-date training.
The identifier and links to information	Lack of consistency between hospitals<br><br>No link to data	Information about diagnosis needs to link to alert on the Electronic Patient Records (EPR).<br><br>Linking primary care records with hospital records such that if a person with dementia presents to hospital, specialist dementia service is notified.<br><br>Critical information should be presented succinctly (not 4-5 pages).
Information	Information should be relevant, succinct, and easy to access	Include key pieces of information about the person (preferences for name, physical contact, food, Lasting Power of Attorney (LPA), Do Not Resuscitate (DNR), etc.) and ways to contact family and carers.<br><br>Patient profile documents would benefit from a redesign towards presenting key information in a succinct way.
Family participation	Input of family is vital<br><br>Involving family to establish patient preferences and legal provisions.	Routinely contacting family and carers, especially when contact with patient is difficult to avoid making assumptions about legal provisions (e.g. Do Not Resuscitate orders).
Standardisation	Ensuring standard of care	Expectation that patients receive similar levels of care irrespective of which hospital they stay at.

Table 2. Themes, subthemes and key points identified from workshop analysis.

For peer review only

Training

Participants often mentioned the need for regular professional standards training. Of particular concern was high staff turnover and the challenges with ensuring consistency of understanding and communication, including using positive language when discussing patients with dementia, across both regular and bank staff. The need for training also applied to other non-clinical staff, such as porters. Carers emphasised the importance of institutional leadership and organisational culture. They also highlighted that experiential training sessions can be more effective for staff, as healthcare professionals are more likely to retain the information if they are learning by doing. There was consensus that the training needed to be of sufficient quality – recognising that mandatory training, especially online, can be of poor quality.

Linking the identifier to key personal information

Participants pointed out the problems arising from an individual's key personal information not accompanying them around various healthcare settings. Some saw the solution in technology, such as smart-linking key personal information to the identifier (e.g., through a QR or bar code), to make it readily available at each point in the care pathway. Some participants saw value in relevant summary information about the patient also being available in a physical form, suggesting that there may be situations where staff might not have time or opportunity for an extra step of scanning a code or checking the electronic patient record.

Integration and management of records

Participants often mentioned that key medical and personal information often exists in separate electronic and paper-based systems which are not integrated. Participants felt that VIS could be instrumental to much better information integration and management along the whole patient care pathway, to include key search functions, automated alerts and links to that individual's care support needs. Again, many suggested that technology may offer useful solutions, from wearables to individual devices with screens at patient bedside.

Enabling carers and families to provide key information

Participants highlighted the need for up-to-date, readily available, accessible and prioritised key information about each hospitalised individual with dementia, e.g., preferred name, food preferences, allergies, ability to communicate and/or give consent, Lasting Power of Attorney, and Do Not Resuscitate orders, to enable appropriate, personalised care. Relatives and carers felt, where possible, they should be enabled to input and update key personal information and emphasised the importance of being able to connect to their family member via a video call.

Standardising the identifier

Perhaps the most contentious issue to arise relating to the identifier was what participants referred to as "standardisation". Many workshop participants considered that a standardised, nationally mandated identifier, applied on admission with assumed consent (unless revoked), might provide greater consistency in recognising patients with a confirmed diagnosis of dementia across different healthcare organisations, particularly given staff mobility across different sites using different systems. However, workshop participants who were healthcare professionals had concerns about how difficult it would be to implement a unified system across the whole NHS given that different VIS are already in existence.

DESIGN PRINCIPLES

Plsek et al. state that '[d]esign rules are heuristic statements in the form: If you want to achieve outcome Y in situation S, something like X might help.'(22) Our approach to deriving design principles (using the term 'principles' in preference to 'rules') from the Phase 1 and 2

For peer review only

thematic analysis was adapted from their concept, which ‘helps convert the tacit knowledge of organizational change agents into explicit, actionable knowledge’.(22) We used an adapted version of their methods 3 (‘listen to stories of change efforts told by change leaders, operational managers, and front-line staff and then extract design rules off-line [via review of transcripts or notes]) and 4 (‘pose hypothetical scenarios to those experienced in organizational change, ask them to “think aloud” regarding how they would approach the situation, and then extract design rules off-line’) to extract from our findings, through a number of iterations, a set of five draft principles for the joint phase 3 workshop. We provided these in concise form to all participants in a single sheet, which we supplemented with additional explanations at the workshop with further expansion on the principles. We invited participants to comment on individual principles in small breakout groups and at plenary sessions. We asked: ‘If these principles were adopted, would: hospital staff recognise people with dementia more quickly; respond to their needs more readily; and provide better, more personalised care?’ BF, NS and AM took notes of the discussion.

In the phase 3 workshop and in the feedback from the members of the Expert Collaborative Group (ECG), participants generally agreed that some of the principles would be more problematic to implement than others, e.g., improved IT systems where the various clinical records, such as from hospital, GP, care home and memory clinic records, could be better integrated with readily accessible key personal information. The point was also made that relying purely on electronic records was risky and that supplementary paper-based information would be essential. When asked which of the principles would be more fundamentally important to implement, phase 3 participants debated two in particular: training (contingent on organisational culture and leadership) and the form of the identifier (a wristband, symbol etc.).

Opinions were divided over which principle was the most important. Some participants thought that a national, standardised identifier would be relatively easy to implement, especially with third-sector support for dissemination. However, as pointed out by members of the ECG given how well-established some systems already are, a single nationally mandated identifier would be difficult, if not impossible, to implement. With regards to training, inadequately trained staff and lack of an ongoing training programme for all patient-facing employees, particularly for new or agency staff, could lead to them not being able to interpret the identifier in a way that supports patients as individuals. On a number of occasions, training was referred to as the ‘bedrock principle’.

A key point from both the phase 3 workshop and the subsequent ECG discussion highlighted the interdependency of the principles which, if adopted singularly and piecemeal, would be unlikely to achieve the goal of more consistent, personalised care for hospitalised people with dementia. In other words, these principles need to be implemented systemically and as a set, if their benefits are to be realised. As a result, we added the interdependency of principles as a further principle. The feedback we received from phase 3, the ECG, and the project partners helped refine further our principles. The challenge involved making the principles simple yet adaptable so that they can serve a large, varied population in the most effective way possible. We provide a summary of the six working principles we arrived at through the phase 3 workshop in Table 3.

For peer review only

DESIGN PRINCIPLES
**1. The hospital trust provides a professionally trained workforce and an appropriate culture of care.**
**2. The symbol is easily recognisable and well understood.**
**3. Key personal information is readily available and accessible.**
**4. Key personal information is integrated into the Electronic Patient Record (EPR).**
**5. Relatives and carers are involved in providing key information and monitoring care.**
**6. The principles need to function as a system to be successful.**

Table 3. The set of 6 design principles resulting from the study.

DISCUSSION

Using an experience-based co-design approach

Whereas there are many variations in the design of VIS, we believe this has been the first attempt to develop a set of high-level principles to guide the design and use of VIS, through a participative co-design approach. Our study shows that designing and running a programme of online workshops using scenario-based narratives is feasible and that these may reduce some of the time and cost while improving accessibility in comparison to in-person workshops. Presenting narratives on slide shows with voice-overs can effectively replace videoed witness accounts typically used in experience-based co-design studies. The experience-based co-design approach can also be used in evaluation studies focused on abstract, high-level design, such as developing principles for visual identification systems.

Reconciling abstract principles with practical reality

In phases 1 and 2, we focussed on the more tangible elements of the VIS – the identifier and the key information and how these might be made to work better, which in turn provoked responses about issues concerning the less visible elements, leading to our findings. We acknowledge that formulating our findings into principles using Plsek et al.'s heuristic was challenging for us (requiring several iterations) and that these were not fully resolved in their initial form in the phase 3 workshop. None-the-less, these principles served as a means of engaging participants in a more abstract level of discussion, along the lines of 'If we were to propose a set of design principles such as these, what would be your response be?'. Participants readily engaged with this more abstract form of discussion.

The value of the phase 3 and Expert Collaborative Group feedback to our draft principles was to reveal the 'sticky materiality of practical encounters',(23) i.e. the potential gap between high-level principles and the friction-laden character of situations on the ground, as well as the need for designing around what happens in reality of how identifiers for people with dementia are used in practice. Therefore, the sixth principle, emphasising the need for the first five principles to be implemented simultaneously is crucial. Discussing draft principles also highlighted the importance of a participatory process, whereby a proposed improvement, in prototype form (in this case our draft principles), can be interrogated and iteratively refined.

For peer review only

Broadening the understanding of an effective VIS

While the two elements – the identifier and some form of document holding key personal information – are undeniably important, it became clear that for a VIS to function effectively, other elements – as identified in our findings – play a vital role in a functioning of the system that benefits the patient with dementia. For instance, lack of training may mean that even though a symbol is used for a hospitalised patient with dementia, some staff members may not be able to interpret it as a prompt. Involving carers in providing key information and monitoring care can help ensure that staff are able to adjust their care to the needs of each patient (e.g., using preferred name or assisting with meals) rather than to exclusively rely on their broad training.

Strengths and limitations

The EBCD approach is adaptable and can be used to promote discussion of abstract high-level service principles as well as identify practical improvements to service design and delivery. Designing and running a programme of online workshops using scenario-based narratives is a feasible and accessible alternative to holding in-person workshops.
The EBCD structure helps bonding between researchers and participants, in turn allowing for insightful and richer discussions, and for developing consensus. The study would have benefited from a broader representation of healthcare professionals including, e.g., ward managers, health care assistants, out-patients' department staff, and peripatetic staff such as porters, as well as people with dementia and their carers.

CONCLUSION

Our study shows that an adapted co-design approach using scenarios can trigger useful discussions with staff and carer groups, identify problems with VIS, and engage participants in an abstract discussion about high-level design principles; and how these might guide the improvement of VIS.(20) Equally importantly, there was a consensus that the efficiency of the principles relied on their interdependent functioning. If only some principles are adopted singularly and piecemeal, this could lead to inconsistent, less personalised care. Thus, these principles need to be implemented systemically and as a set, if their benefits are to be realised. While we recognise these principles are not yet fully resolved, we have made progress towards better defining these principles. Constraints on the normal experience-based co-design approach imposed by the linked-study commissioning method and the pandemic proved an initial challenge; ultimately, they were not an impediment to creatively developing an effective approach. This method could potentially be applied to other forms of service evaluation and improvement initiatives.

Acknowledgements

We acknowledge the helpful feedback, at all stages in our study from Graham Martin, Director of Research at THIS Institute, and at key points, from our Expert Collaborative Group. Actor Francesca Isherwood provided the voice-overs for the Phase 1 materials. Joanna Goodrich presented the study with NS to the HSRUK conference in Sheffield in July 2022. We gratefully acknowledge the participation of the carers and staff in the workshops, and members of the Expert Collaborative Group for their constructive input. We are grateful to the charity Dementia Carers Count for connecting us with the carers included in this study. The icons in figures 1 and 2 are used by kind permission of Flaticon.com.

Contributor Statement

BF, NS and AM were the researchers, led by BF, comprising the co-design study and all contributed to the drafting of this manuscript, led by AM. KK, reviewed and substantially edited and contributed to the manuscript. All authors reviewed the final version.

For peer review only

Competing Interests

The authors declare no conflict of interest. The funders had no role in the design of the study; in the collection, analyses or interpretation of data; in the writing of the manuscript, or in the decision to publish the results.

Funding

Health Foundation, Grant/Award Number: RHZF/001 - RG88620; Health Services and Delivery Research Programme, Grant/ Award Number: NF-SI-0617-10026

Data Sharing Statement

Not applicable. Hand-written notes only taken for later thematic analysis.

Ethical Approval Statement

As service improvement, this EBCD study did not require an ethics review.

REFERENCES

1. Wye Valley NHS Trust. Forget me not [Internet]. Herefordshire Council; 2018 [cited 2022 Sep 8]. Available from: https://www.wyevalley.nhs.uk/visitors-and-patients/county-hospital-(acute)/in-patients/dementia-care/forget-me-not-1.aspx

2. The Butterfly Scheme. The Butterfly Scheme | Reaching out to people with dementia and to anyone needing memory support [Internet]. 2017 [cited 2022 Sep 8]. Available from: https://butterflyscheme.org.uk/

3. East Sussex Healthcare NHS Trust. This is me care passport [Internet]. 2013 [cited 2022 Sep 8]. Available from: https://www.esht.nhs.uk/wp-content/uploads/2017/10/This-is-me-My-Care-Passport.pdf

4. Alzheimer's Society. This is me [Internet]. 2022 [cited 2022 Sep 8]. Available from: https://www.alzheimers.org.uk/get-support/publications-factsheets/this-is-me

5. Professional Records Standards Body. About me [Internet]. 2022 [cited 2022 Sep 8]. Available from: https://theprsb.org/standards/aboutme/

6. Kuberska K, Dixon-Woods M, Martin G. Visual identifier systems for patients with cognitive impairment in healthcare settings: a survey of practice in UK hospitals. International Journal of Older People Nursing. 2022;

7. Royal College of Psychiatrists. National Audit of Dementia Care in General Hospitals 2018–2019. Round Four Audit Report. London: Royal College of Psychiatrists; 2019 p. 105.

8. THIS Institute. Developing a visual identification method for people with cognitive impairment in institutional settings [Internet]. THIS Institute - The Healthcare Improvement Studies Institute. 2020 [cited 2022 Sep 8]. Available from: https://www.thisinstitute.cam.ac.uk/research-projects/davinci/

9. Featherstone K, Boddington P, Northcott A. Using Signs and Symbols to Label Hospital Patients with a Dementia Diagnosis: Help or Hindrance to Care? Narrative Inquiry in Bioethics. 2020;10(1):49–61.

10. Brigden T, Mitchell C, Ordish J, Hall A. Visual identifiers in the care of people with dementia: An ethical and legal analysis [Internet]. Cambridge: PHG Foundation; 2020

For peer review only

[cited 2022 May 9]. Available from: https://www.phgfoundation.org/media/44/download/visual-identifiers-in-the-care-of-people-with-dementia.pdf?v=1&inline=1

11. Sutton E, Armstrong N, Locock L, Conroy S, Tarrant C. Visual identifiers for people with dementia in hospitals: a qualitative study of a classification system for improving quality of care. BMJ Quality & Safety. forthcoming;

12. Donetto S, Pierri P, Tsianakas V, Robert G. Experience-based Co-design and Healthcare Improvement: Realizing Participatory Design in the Public Sector. The Design Journal. 2015 Jun 1;18(2):227–48.

13. Point of Care Foundation. 1. What is Experience-based co-design? [Internet]. Point of Care Foundation. 2021 [cited 2023 Feb 9]. Available from: https://www.pointofcarefoundation.org.uk/resource/experience-based-co-design-ebcd-toolkit/step-by-step-guide/1-experience-based-co-design/

14. Point of Care Foundation. EBCD: Experience-based co-design toolkit [Internet]. Point of Care Foundation. n.d. [cited 2022 Sep 8]. Available from: https://www.pointofcarefoundation.org.uk/resource/experience-based-co-design-ebcd-toolkit/

15. Goodrich J. Why experience-based co-design improves the patient experience. The Journal of Health Design. 2018 Mar 22;3(1):84–5.

16. Springham N, Robert G. Experience based co-design reduces formal complaints on an acute mental health ward. BMJ Open Quality. 2015 Jan 1;4(1):u209153.w3970.

17. Brady GC, Goodrich J, Roe JWG. Using experience-based co-design to improve the pre-treatment care pathway for people diagnosed with head and neck cancer. Support Care Cancer. 2020 Feb;28(2):739–45.

18. Locock L, Robert G, Boaz A, Vougioukalou S, Shuldham C, Fielden J, et al. Using a national archive of patient experience narratives to promote local patient-centered quality improvement: an ethnographic process evaluation of "accelerated" experience-based co-design. J Health Serv Res Policy. 2014 Oct;19(4):200–7.

19. Dunne A, Raby F. Speculative everything: design, fiction, and social dreaming. Cambridge, Massachusetts ; London: The MIT Press; 2013. 224 p.

20. INVOLVE, NIHR. Policy on payment of fees and expenses for members of the public actively involved with INVOLVE [Internet]. 2016 [cited 2022 Sep 8]. Available from: https://www.invo.org.uk/wp-content/uploads/2016/05/INVOLVE-internal-payment-policy-2016-final-1.pdf

21. Ramanadhan S, Revette AC, Lee RM, Aveling EL. Pragmatic approaches to analyzing qualitative data for implementation science: an introduction. Implement Sci Commun. 2021 Jun 29;2(1):70.

22. Plsek P, Bibby J, Whitby E. Practical Methods for Extracting Explicit Design Rules Grounded in the Experience of Organizational Managers. The Journal of Applied Behavioral Science. 2007 Mar 1;43(1):153–70.

23. Tsing AL. Friction: An Ethnography of Global Connection. Princeton: Princeton University Press; 2005. 336 p.

For peer review only

FIGURE LEGENDS

Figure 1. The introductory slide from the stage 1 workshop to explain the concept of the Visual Identification System with two key elements (within the blue circle) and how these relate to other relevant aspects of the hospital care environment.

Figure 2. A further slide from the stage 1 workshop illustrating key components of the Visual Information System for a fictional patient 'Alice', i.e., the 'visual identifier' and the 'information element'.

For peer review only

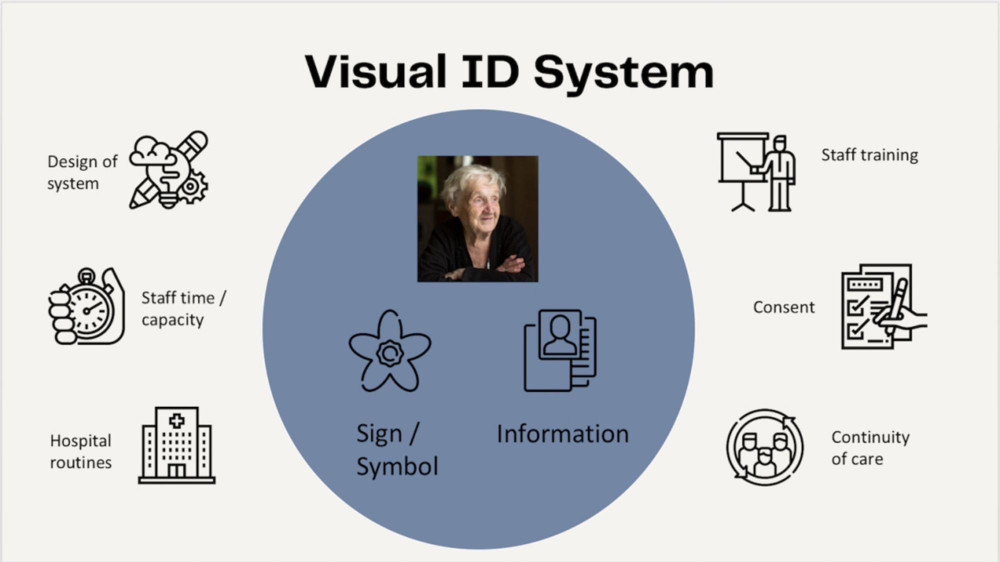

Figure 1. The introductory slide from the stage 1 workshop to explain the concept of the Visual Identification System with two key elements (within the blue circle) and how these relate to other relevant aspects of the hospital care environment.

119x67mm (300 x 300 DPI)

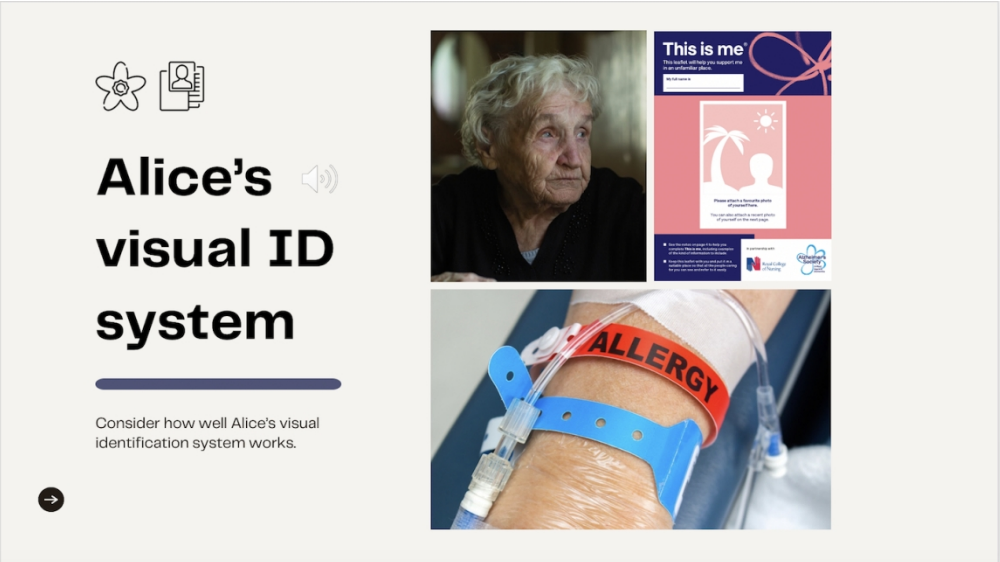

Figure 2. A further slide from the stage 1 workshop illustrating key components of the Visual Information System for a fictional patient 'Alice', i.e., the 'visual identifier' and the 'information element'.

119x67mm (300 x 300 DPI)

PHASE 1 DISCOVERY WORKSHOP	
**Titles of scenario slides**	**Content and questions delivered via actor voice-over**
**Introduction**	Explanation of Visual Identification Systems (VIS)
**Alice's Story**<br>**Alice's VIS**<br>**Alice's hospital**	*What information about Alice is needed to:*<br>• *keep her safe while she is in hospital?*<br>• *help staff communicate well with her?*<br>• *make sure she gets the right treatment and care?*<br>• *avoid agitation and distress?*<br>• *make sure she is treated like a person?*
**Consenting to the identifier**<br>**Issues of Consent**	• *Should patients/relatives give consent for an identifier to be used? If so, what should the care team do to get consent?*<br>• *What do patients/relatives need to know about the implications of having an identifier?*
**George's story**<br>**George's visual IDs**<br>**Continuity of care**	• *How can we make sure that it is clear to all hospital staff what the identifier means, and what they should do when they see it?*<br>• *How can we ensure essential information is easy to find and that all staff know about and consult the information?*
**Claire's story**<br>**Claire's hospital**<br>**Visual identifiers**	• *Given their busy schedules, how can we ensure staff have the time to consult the patient information documents?*<br>• *How can we better ensure that appropriate care continues from one shift to the next?*
**What did we miss?**	• *What did we miss?*

For peer review only

PHASE 2 CO-DESIGN WORKSHOP	
**Scenario slides**	**Content and questions delivered verbally by researchers**
**Recap/intro: VIS**<br>**How it should work**<br><br>**The sign/symbol element**<br>**The information element**<br><br>**How do we overcome these obstacles?**	
**6 hat thinking**	
**The sign / symbol**	• *What should the symbol tell you about the individual?*<br>• *Do different systems across different sites lead to problems?*<br>• *What might make it better?*
	• *What if the symbol was the wristband (perhaps coloured), was personalized and smart in some way - like a unique RIFD, tracking, QR or barcode?*<br>• *What would be the advantages of this approach?*<br>• *What would make this work well?*
**The information (1)**	• *Could a standardised tool be used as part of the admissions process?*<br>• *How best might this information be gathered?*
**The information (2)**	• *What is the key information that is needed to allow them to be treated both as a person and for their specific dementia and other care needs?*<br>• *Would it be best if the essential information about the patient is stored and managed as part of the EPR system, rather than separately stored information?*
**Making the system work (1)**	• *Who would have responsibility for completing this?*<br>• *How could we make sure it is absolutely clear whose responsibility it is to enter the information and that it gets done?*<br>• *How can we make sure that all staff know about, can find, consult and act on this information?*
**Making the system work (2)**	• *Assuming the details are on the system, how would it benefit the bedside care of people with dementia?*<br>• *How can we make this system work when the patient is moved away from the bedside?*
**What did we miss?**	• *What did we miss?*

For peer review only

PHASE 3 VERIFICATION WORKSHOP	
**Stage**	**Content and questions delivered verbally by researchers**
**Welcome and introductions (Plenary)**	
**Recap on progress made in prior workshops (Plenary)**	
**Introduction to the design principles (Plenary)**	• What MUST happen, or MUST be in place, for the system to work so that:<br>1. hospital staff recognise people with dementia more quickly<br>2. respond to their needs more readily<br>3. and provide better, more personalised care
**Sense-checking the design principles (Plenary)**	• Do you have any questions about the principles?<br>• What did you think of the idea of a set of five co-dependent principles?
**Critiquing the design principles (Small mixed groups)**	• Which principle(s) do you most strongly agree with? Why?<br>• Are there any principles on the list that you wouldn't necessarily include? Why?<br>• Are there any missing from the list?
**Feedback (Plenary)**	
**How to implement the design principles (Small mixed groups)**	• How viable is it to implement any/some/all of these principles?<br>• If so, what would need to happen first?<br>• What would help implement them?<br>• What would get in the way?
**Feedback (Plenary)**	
**Anything we missed? (Plenary)**	• *What did we miss?*

For peer review only
